# Correlations between molecular structure and biological activity in "logical series" of dietary chromone derivatives

**DOI:** 10.1371/journal.pone.0229477

**Published:** 2020-08-21

**Authors:** Włodzimierz Lewandowski, Hanna Lewandowska, Aleksandra Golonko, Grzegorz Świderski, Renata Świsłocka, Monika Kalinowska

**Affiliations:** 1 Department of Chemistry, Biology and Biotechnology, Bialystok University of Technology, Bialystok, Poland; 2 Institute of Nuclear Chemistry and Technology, Centre for Radiation Research and Technology, Warsaw, Poland; 3 Department of Microbiology, Institute of Agricultural and Food Biotechnology, Warsaw, Poland; Babasaheb Bhimrao Ambedkar University (A Central University), INDIA

## Abstract

The research was conducted in the “logical series” of seven ligands: chromone, flavone, 3-hydroxyflavone, 3,7-dihydroxyflavone, galangin, kaempferol and quercetin. Each subsequent ligand differs from the previous one, among others by an additional hydroxyl group. The studied chromone derivatives are plant secondary metabolites which play an important role in growth, reproduction, and resistance to pathogens. They are important food ingredients with valuable pro-health properties. The studies of the relationships between their molecular structure and biological activity facilitate searching for new chemical compounds with important biological properties not by trial and error, but concerning the impact of specific changes in their structure on the compound properties. Therefore several pectroscopic methods (FT-IR, FT-Raman, ^1^H and ^13^C NMR) were applied to study the molecular structure of the compounds in the series. Moreover the quantum-chemical calculations at B3LYP/6-311++G** were performed to obtained the theoretical NMR spectra, NBO atomic charge, global reactivity descriptors and thermodynamic parameters. The antioxidant activity of the compounds was tested in the DPPH and FRAP assays and the mechanism of antioxidant activity was discussed based on the results on theoretical calculations. The cytotoxicity of the ligands toward human epithelial colorectal adenocarcinoma Caco2 cells was estimated and correlated with the lipophilicity of the compounds. The principal component analyses (PCA) and hierarchical cluster analysis were used to study the dependency between the molecular structure of ligands and their biological activity. The experimental data were related to the theoretical ones. The found regular changes in physicochemical properties correlated well with the systematic changes in antioxidant and biological properties.

## Introduction

Chromone (1-benzopyran-4-one) is the backbone of many flavanols, flavones, isoflavones and flavonoids found in the plant kingdom and dietary products. Chromones are active in various plant cycles, among others in growth regulation and stimulation of oxygen uptake [1]. The chromone structural system is used to synthesize many anti-HIV, anti-inflammatory, antibacterial and anticancer drugs as well as those used in neurodegenerative diseases, inflammatory diseases and diabetes [1]. Investigations of this class of compounds showed that they have promising properties as inhibitors of tyrosine kinases, carbonate anhydrases, NF-κB factor, sirtuins, topoisomerases, adenosine A3 receptors, as well as modulators in Keap1-Nrf2 signaling pathway. Interestingly, chromone-metal complexes of Pd(II), Cu(II), Ru(II), Ni(II) show relatively high cytotoxicity in many neoplastic cell lines. Current reports confirm that cytotoxicity of such structures is correlated with their shape, size, polarizability, electrical state and dipole moment, which additionally justifies the advisability of conducting chemical modifications of chromone derivatives in the design of new pharmacological agents based on natural products [2]. Chromones are compounds capable of scavenging many types of radical oxygen species (ROS) and inhibiting lipid peroxidation. Scavenging of free radicals can occur through rapid hydrogen transfer. In this regard, the high reactivity of hydroxyl substituents influences to the greatest extent the antioxidant capacity of a given compound [3]. Electron transfer and the stability of the corresponding phenoxyl radicals resulting from H-abstraction also play an important role in this process. In fact, the total antioxidant capacity and redox potential are defined by several conformation factors (substitution pattern of functional groups including hydroxyl groups), phenoxyl radical stabilization and electron delocalization in the molecule (energy of the HOMO and the LUMO molecular orbital) [4].

**Fig 1:**
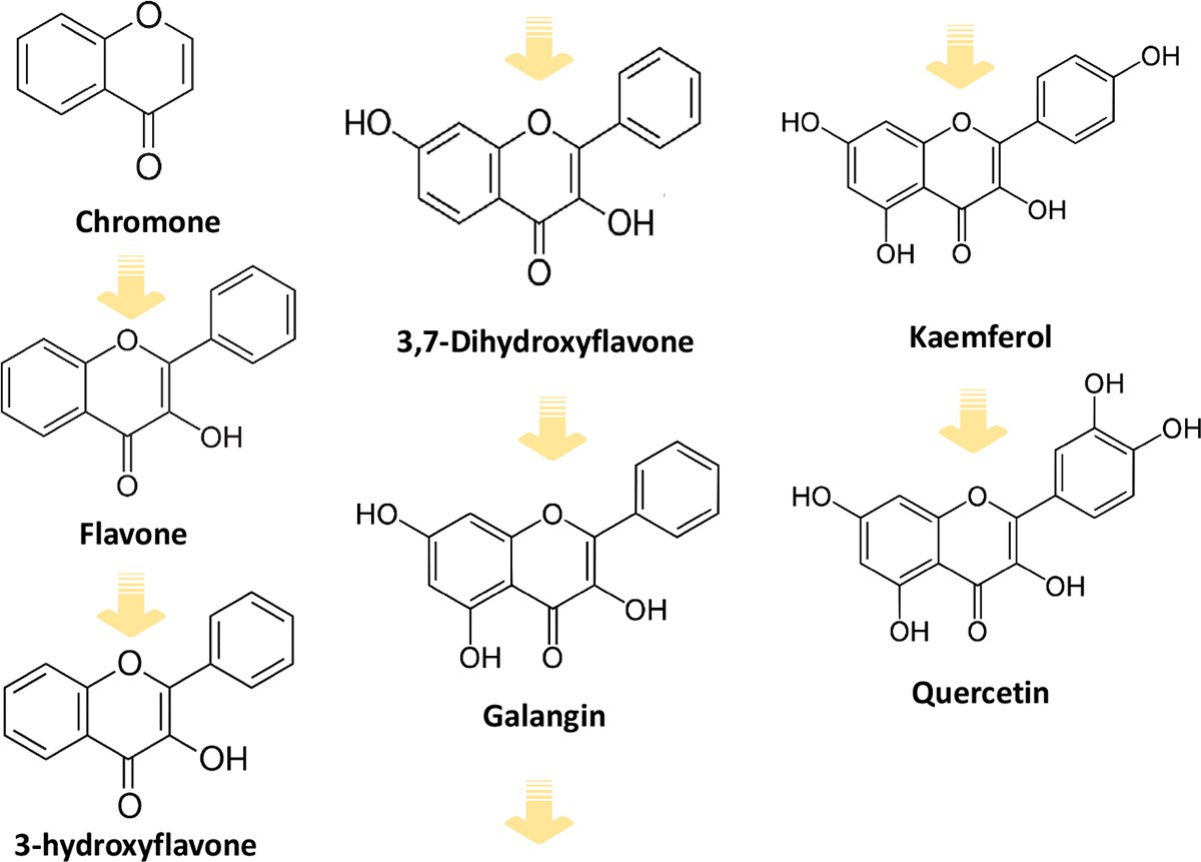
The concept of a logical series of seven ligands.

Even a small change in one of the mentioned factors may cause significant differences in the physicochemical and biological properties of the compound. Therefore in this paper, the relationship between the structure and the biological properties of six chromone derivatives i.e. flavone, 3-hydroxyflavone, 3,7-dihydroxyflavone, galangin, kaempferol, quercetin (Fig 1) has been investigated. Flavone forms a heterocyclic system that is the basic structure of flavonoids. 3-hydroxyflavone is a synthetic compound not found naturally in plants. It has fluorescent properties. The analogues of this compound are promising pharmaceuticals for the treatment of diabetes and insulin resistance by increasing the uptake of glucose by skeletal muscle [5]. 3,7-Dihydroxyflavone has a high potential for the treatment of respiratory tract infections (*Staphylococcus pneumoniae*), and its derivatives inhibit the growth of tumour cells *in vitro*. Galangin occurs e.g. in *Alpinia officinarum Hance* and propolis. It has antibacterial and antiviral properties. It inhibits the growth of human breast cancer cells *in vitro* [6]. Galangin has a bacteriostatic effect on *Staphylococcus aureus* strains, both sensitive to antibiotics and methicillin-resistant [7]. Kaempferol is present in tea leaves, blackthorn flowers, wildflowers. It occurs most often as a glycoside aglycon. This compound has anti-proliferative, pro-apoptotic effects on lung, breast, pancreas, stomach and other cells. The mechanisms of its action include apoptosis, cell cycle arrest at the G2/M phase, downregulation of epithelial-mesenchymal transition (EMT)-related markers, and phosphoinositide 3-kinase/protein kinase B signaling pathways [8]. Kaempferol has been shown to exhibit strong cellular antioxidant ability. Pretreatment with kaempferol significantly attenuated the ROS-induced hemolysis of human erythrocytes (87.4% hemolysis suppressed at 100 μg/mL) and reduced the accumulation of toxic lipid peroxidation product malondialdehyde (MDA) [9]. The anti-hemolytic activity of kaempferol was mainly through scavenging of excessive ROS and preserving the intrinsic antioxidant enzymes (superoxide dismutase, SOD; catalase, CAT; and glutathione peroxidase, GPx) activities in normal levels [9]. Quercetin was shown to modulate many enzymatic pathways, including cyclooxygenase, lipoxygenase and tyrosine kinase-dependent ones. It shows anti-inflammatory activity by inhibiting the release of histamine, pro-inflammatory cytokines (including IL-4) and leukotrienes production [10]. Quercetin is a flavonoid that is the scaffold or many other flavonoids such as rutin, hesperidin, naringin, tangeritin.

Several papers are dealing with the chemical structures and biological activity of these compounds [7, 8, 11, 12]. Still, there are few studies on the relationship between their molecular structure and biological activity. The number and position of hydroxyl substituents in the rings of molecules influence the distribution of electronic charge in a molecule and thus induce changes in its polarity, lipophilicity and acceptor-donor capacity. All these factors have a significant impact on the biological activity of these compounds, including cell membrane permeability and the antioxidant capacity.

The knowledge acquired relationship between the molecular structure and electronic charge distribution in molecules of natural origin is the foundation for the precise design of drugs, food additives or plant protection products. All groups of compounds that will ultimately be used in contact with living organisms are characterized in detail in terms of their toxicity and bioavailability, hence it is so important to know the impact of small changes in their structure on the biological effect. In addition, knowing the structure-activity relationships, they can be modified in a targeted and precise manner to meet the expected function as much as possible. In this article, we focused on the search for correlations between selected physicochemical, structural and biological descriptors of popular plant metabolites with a unique chemical structure ordered into a logical structural sequence. Therefore the molecular structure of ligands was characterized by the experimental (FT-IR, FT-Raman, ^1^H and ^13^C NMR) as well as theoretical parameters calculated at B3LYP/6-311++G** level and then was discussed in term of their biological (antioxidant, cytotoxic and lipophilic) activities.

## Materials and methods

### Materials and spectroscopic characterization

Chromone and its derivatives, DPPH (2,2-diphenyl-1-picrylhydrazyl), 2,4,6-tripyridyl-s-triazine (TPTZ), FeCl_3_·6H_2_O, FeSO_4_ were purchased from Sigma-Aldrich Co. (St. Louis, MO, USA) and used without purification. Methanol was purchased from Merck (Darmstadt, Germany).

The FT-IR spectra were registered in KBr matrix pellets on Alfa Bruker spectrometer (Bremen, Germany) within the range of 400–4000 cm^-1^ with the resolution of 2 cm^-1^. The Raman spectra were recorded in the range of 100–4000 cm^-1^ with the MultiRam Bruker spectrometer (Bremen, Germany) with the resolution of 1 cm^-1^. The ^1^H (400.15 MHz) and ^13^C (100.63 MHz) spectra were registered with the Bruker Avance II 400 spectrometer (Bremen, Germany) in DMSO-D6 solution. TMS was used as an internal reference.

### Evaluation of biological activity

#### Antioxidant activity assays

The antiradical activity was conducted according to the DPPH assay described in [13]. The initial methanolic solutions of reagents were prepared at the concentration of 500–50 μM and 60 μM for DPPH. Appropriate volumes of tested compound solutions were added into the test tubes and diluted with methanol to obtain a series of solutions with different concentrations (final volume 1 mL). Then, 2 mL of DPPH was added to each tube, vortexed and incubated in the darkness for 1 h at 23°C. The final concentration of DPPH was 40 μM. The absorbance of the mixture was measured at 516 nm against methanol as the blank using Agilent Carry 5000 spectrophotometer. The control sample—2 mL of DPPH solution and 1 mL of methanol. The antiradical activity of compounds against DPPH radical was calculated according to the equation:

$$\%\;I=\frac{A_{control}^{516}-A_{sample}^{516}}{A_{control}^{516}}∙100\%$$

where % I—% inhibition of DPPH radical, A^516^_control_—absorbance of the control, A_516_ sample—absorbance of the sample. Then, the concentration of the tested substances was plotted against the % inhibition and the EC_50_ values were read from the scavenging curves. The EC_50_ parameter means the concentration of a substance that inhibits 50% of the radical.

Ferric reducing antioxidant activity was determined in the FRAP assay [14]. To prepare the FRAP reagent 0.3 M acetate buffer (pH 3.6), 10 mM TPTZ (in 40 mM HCl) and 20 mM FeCl_3_·6H_2_O (in water) were mixed in a volumetric ratio 10:1:1 directly before analyses. Then, the FRAP reagent (3 mL) was mixed with the tested substance (0.4 mL; final concentrations C = 20 mM). The absorbance was measured at 594 nm against a blank (3 mL of FRAP and 0.4 mL of methanol) using an Agilent Carry 5000 spectrophotometer (CA, USA). Antioxidant activity was expressed as Fe^2+^ equivalents [μM] using the calibration curve prepared over the range of 200–0.1 μM concentration of FeSO_4_.

#### Neutral red (NR) uptake assay of cell metabolic activity

Human epithelial colorectal adenocarcinoma Caco-2 cells were purchased from ATCC and cultured in EMEM medium containing 20% FBS and Pen-Strep, and grown under standard conditions. Cells in the logarithmic phase were seeded in polylysine-coated 96-well culture plates and cultured at 37°C under a 5% CO_2_ atmosphere for 24 hours. The initial number of cells per well was 5 000. The cultured medium was removed when the cells adhered to the plate wall. The cells were then incubated in 100 μL of medium with the growing concentrations of the studied compound, for 48 h. Non-treated cells were used as control. After this time, 50 uL of the medium containing NR was added to the final concentration of 3 mg/mL NR and incubated for 2.5 h. Then, the dye-containing medium was removed, cells were washed with PBS and lysed in 50% EtOH, 1% AcOH and 49% water mixture (20 min, shaking). Fluorescence was measured at Ex/Em 530/645 nm respectively, in a plate reader spectrophotometer Infinite M200 (Tecan, Austria). IC_50_ values were calculated by fitting Hill equation-based logistic curves.

### Computational details

The geometries of the studied molecules and their radicals, radical cations and anions were calculated using DFT method with B3LYP functional and the 6–311++G** basis set in the gas phase, water and methanol solvents using Gaussian 09W program package [15]. For the solvent optimization the IEF-PCM method was used. Vibrational frequencies were computed to ensure no imaginary frequency for the optimized structures. For the neutral molecules in the gas phase, the NBO atomic charges were calculated. The ^1^H and ^13^C NMR spectra were performed in DMSO solvent using GIAO formalism. The highest occupied molecular orbital (HOMO) and lowest unoccupied molecular orbital (LUMO) and the energy difference between them (energy gap, ΔE) were calculated. The global reactivity descriptors: electronegativity, electrophilicity index, hardness, softness etc. are calculated for the molecule. The BDE, IP, PDE, PA and ETE parameters were calculated in the gas phase, water and methanol solution. All enthalpies were calculated for 298.15 K and 1.0 atmosphere pressure. The calculated gas-phase enthalpy for an electron (e), a proton (H^+^), an and hydrogen atom (H^•^) is 3.145 [16], 6.197 [17], and -1306 kJ/mol respectively. For the solvent phase calculation in water, ΔH(hydration) of the electron (e), proton (H^+^) and the hydrogen atom (H^•^) were respectively taken as -105 kJ/mol, -1090 kJ/mol and -4.0 kJ/mol [18, 19] ΔH(solvation) for the electron (e), proton (H^+^) and the hydrogen atom (H^•^) were taken as -86 kJ/mol, -1038 kJ/mol and 5 kJ/mol [18] respectively in methanol. The values of the partition coefficients in octan-1-ol/water solvent system were calculated in ACD/Labs program.

### Statistical analysis

The experimental data were expressed as mean values ± standard error of determinations made in triplicates. Correlations between particular experimental and theoretical data were expressed as correlation coefficient (R) and tested for significance by t-test (P < 0.05). For multifactorial comparison, principal component analyses (PCA) and hierarchical cluster analysis were done. The distances between samples were calculated using Ward’s method and square Euclidean distances. Standardization of the raw data was performed. The dendrogram similarity scales were generated by the Statistica 13.3 program.

## Results

### The antioxidant activity of chromone derivatives

**Fig 2:**
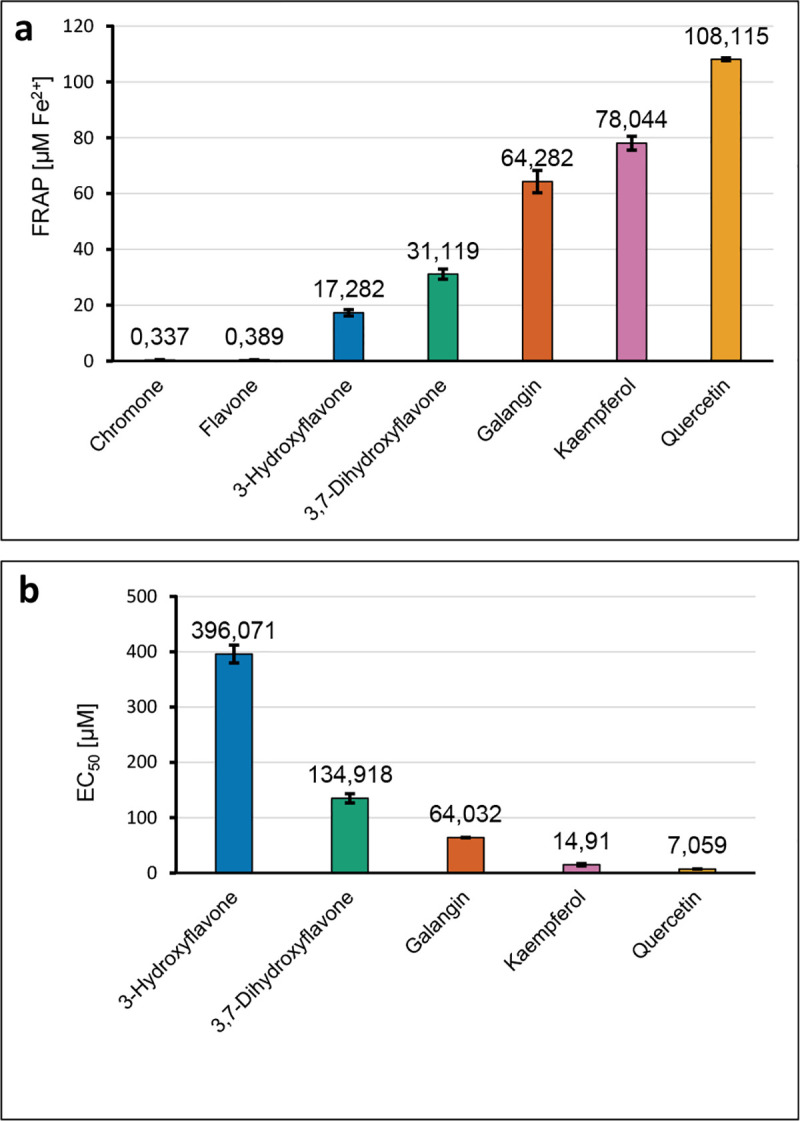
(a) The FRAP values (concentration of tested compounds 20 mmol/dm^3^); (b) the EC_50_ values [μM] obtained in the DPPH assay for the series of ligands.

The antioxidant potential of the compounds was studied by FRAP (Ferric Ion Reducing Antioxidant Parameter) and DPPH (1,1-diphenyl-2-picrylhydrazyl) methods. The first assay is classified as totally SET (single electron transfer), i.e. one electron is transferred from the antioxidant molecule and reduces iron ions bound in the colour complex TPTZ (2,4,6-tris(2-pyridyl)-1,3,5-triazine). The DPPH assay is considered as having a mixed mechanism which depends on the structure of an antioxidant, pH and solvent polarity, i.e.: (a) HAT (hydrogen atom transfer), (b) PCET (proton-coupled electron transfer), (c) SPLET (sequential proton loss electron transfer) and ET-PT (electron transfer followed by proton transfer) [20, 21]. Hydrogen bonding solvents repress hydrogen atom transfer and favor electron transfer, so compounds that are strongly active in hydrogen atom transfer appear to be slower reacting in polar solvents (water, methanol, ethanol) [22]. The obtained results are presented in Fig 2. The lower EC_50_ value the better antiradical activity toward DPPH·, and the higher FRAP values the better ferric reducing antioxidant activity of compounds (which means an increase in the concentration of reduced Fe^3+^ to Fe^2+^). In the FRAP test a regular increase in antioxidant activity was observed in the series chromone → flavone → 3-hydroxyflavone → 3,7-dihydroxyflavone → galangin → kaempferol → quercetin. Analogically in the DPPH assay, a regular increase in the antioxidant activity was observed in the series 3,7-dihydroxyflavone → galangin → kaempferol → quercetin (Fig 2).

### Lipophilicity and cytotoxic activity of chromone derivatives

The calculated lipophilicity parameters are shown in Table 1. The studied compounds are known for their hydrophobicity and poor water solubility. In the series of 3-hydroxyflavone → 3,7-dihydroxyflavone → galangin → kaempferol → quercetin the lipophilicity decreases. Much higher lipophilicity of flavone, 3-hydroxyflavone, 3,7-dihydroxyflavone, and galangin suggests that these compounds may have higher affinities for lipid membranes and have a greater degree of cellular absorption than chromone, kaempferol and quercetin. In Table 2 the cytotoxicity of studied compounds toward human epithelial colorectal adenocarcinoma Caco-2 cells is shown.

**Table 1:** Theoretically and experimentally determined logP values.

Compound	ACD/LogP Classic	ACD/LogP Galas	Experimental	
**Chromone**	1.38±0.43	1.66		
**Flavone**	3.56±0.30	3.38		
**3-Hydroxyflavone**	3.76±0.43	2.39		
**3,7-Dihydroxyflavone**	3.27±0.5	2.42		
**Galangin**	2.83±0.59	2.93		
**Keampferol**	2.05±0.60	2.15	3.11±0.54	[23]
**Quercetin**	2.07±0.72	1.92	1.82±0.32	[23]

**Table 2:** The toxicity of the described series of compounds (IC_50_ [μM]).

Compound	IC_50_±SD [μM]
Chromone	1970±200
Flavone	90±6
3-Hydroxyflavone	28±3
3,7-Hihydroxyflavone	42±3
Galangin	49±6
Kaempferol	167±20
Quercetin	197 ± 20

### DFT study

The calculated, at B3LYP/6-11++G** level, structures of chosen ligands are shown in S12 Table in S2 File The geometrical parameters and NBO atomic charges calculated for the ligands are gathered in S1-S10 Tables of S1 File. In Table 3 the NBO atomic charges for the most stable conformers of studied compounds are shown.

**Table 3:** The NBO atomic charges calculated for studied compounds (conformers with the lowest energy).

		Chromone	Flavone	3-Hydroxyflavone	3,7-Hydroxyflavone	Galangin	Kaempferol	Quercetin
**A**	**C8**	-0.234	-0.234	-0.233	-0.295	-0.329	-0.334	-0.318
	**C9**	0.332	0.337	0.346	0.376	0.391	0.390	0.385
	**C10**	-0.177	-0.174	-0.183	-0.208	-0.270	-0.262	-0.262
	**C5**	-0.177	-0.144	-0.144	-0.122	0.398	0.396	0.403
	**C6**	-0.209	-0.211	-0.213	-0.284	-0.337	-0.337	-0.351
	**C7**	-0.169	-0.170	-0.169	0.347	0.366	0.365	0.366
**Total charge**		-0.634	-0.596	-0.596	-0.186	0.219	0.218	0.223
**C**	**O1**	-0.481	-0.496	-0.481	-0.485	-0.481	-0.484	-0.491
	**C2**	0.222	0.376	0.303	0.311	0.327	0.330	0.314
	**C3**	-0.351	-0.331	0.200	0.210	0.198	0.204	0.208
	**C4**	0.484	0.485	0.455	0.448	0.455	0.438	0.437
	**C10**	-0.177	-0.174	-0.183	-0.208	-0.270	-0.262	0.385
	**C9**	0.332	0.337	0.346	0.376	0.391	0.390	-0.262
**Total charge**		0.029	0.197	0.640	0.652	0.620	0.616	0.853
**B**	**C3’**		-0.204	-0.209	-0.201	-0.203	-0.247	-0.114
	**C4’**		-0.185	-0.188	-0.189	-0.186	0.332	-0.229
	**C5’**		-0.204	-0.207	-0.206	-0.206	-0.285	0.248
	**C6’**		-0.157	-0.151	-0.159	-0.157	-0.134	0.295
	**C1’**		-0.099	-0.097	-0.115	-0.118	-0.147	-0.243
	**C2’**		-0.158	-0.159	-0.174	-0.172	-0.149	-0.160
**Total charge**			-1.007	-1.011	-1.044	-1.042	-0.630	-0.203
	**O1**	-0.481	-0.496	-0.481	-0.485	-0.481	-0.484	-0.491
	**O3**			-0.674	-0.679	-0.676	-0.682	-0.679
	**O4**	-0.580	-0.587	-0.619	-0.634	-0.687	-0.692	-0.688
	**O5**					-0.668	-0.669	-0.668
	**O7**				-0.660	-0.657	-0.658	-0.659
	**O3’**							-0.706
	**O4’**						-0.664	-0.663

e = 1,602*10^−19^ C.

In S1 Fig of S1 File the most stable conformers of studied ligands are ordered with the decreasing value of their energy in relation to the energy of chromone. Addition of one more ring (B) to the chromone backbone causes a decrease in the energy of the molecule by approximately 231 a.u. Interestingly, the substitution of the hydroxyl substituents to the rings causes further stabilization of the molecules and regular decrease in the energy by approx. 75.24–75.26 a.u. in the series of molecules: 3-hydroxyflavone → 3,7-dihydroxyflavone → galangin → kaempferol → quercetin.

**Fig 3:**
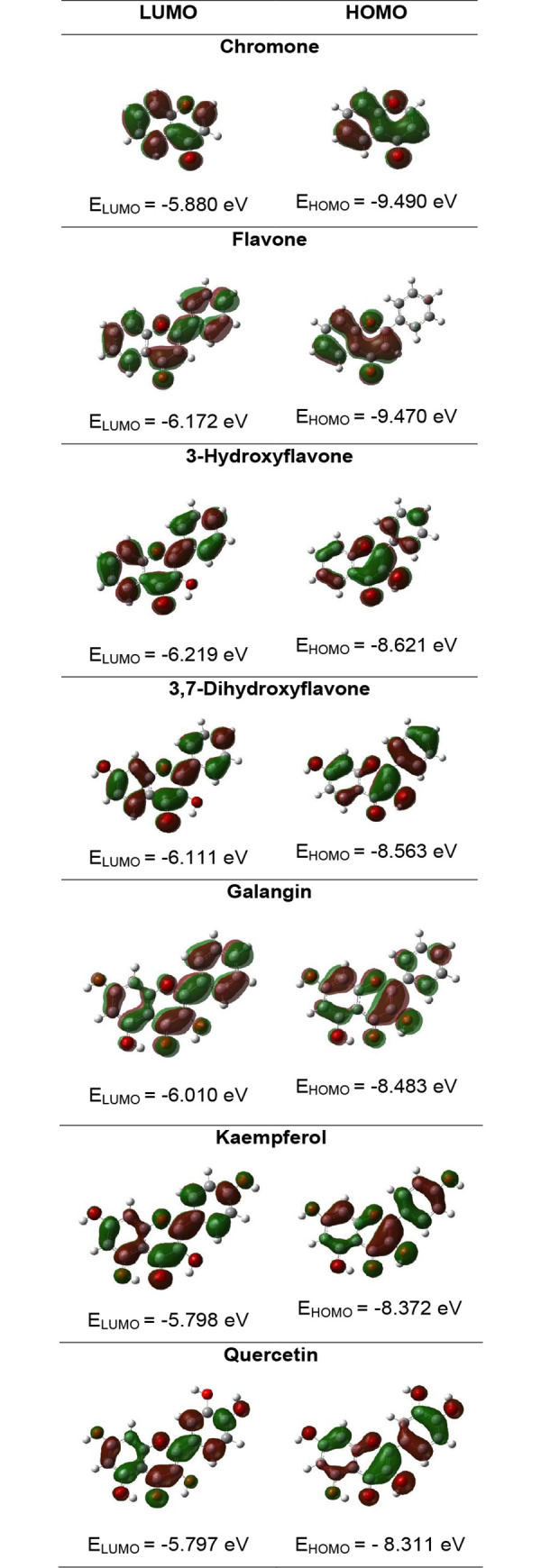
The energy and distribution of LUMO and HOMO orbitals for the studied compounds in the gas phase calculated at B3LYP/6-311++G** level.

In Fig 3 the energy and distribution of the HOMO and LUMO orbitals are shown. In Table 4 the electronic parameters calculated based on the HOMO and LUMO energy values are gathered. Whereas in Table 5 the different parameters related to the specific mechanisms of antioxidant action are listed. The calculations were performed in the gas phase, methanol and water, because the DPPH and FRAP assays were conducted in methanol and water, respectively. The energies of C3-O3· radicals were calculated and compared in the series: 3-hydroxyflavone→3,7-dihydroxyflavone→galangin→kaempferol→quercetin.

**Table 4:** Calculated electronic parameters for the studied ligands at the B3LYP/6-311++G** level.

Parameter	Chromone	Flavone	3-Hydroxyflavone	3,7-Dihydroxyflavone	Galangin	Kaempferol	Quercetin
LUMO (hartree)	-0.216	-0.227	-0.229	-0.225	-0.221	-0.213	-0.213
HOMO (hartree)	-0.349	-0.348	-0.317	-0.315	-0.312	-0.308	-0.305
LUMO (eV)	-5.880	-6.172	-6.219	-6.111	-6.010	-5.798	-5.797
HOMO (eV)	-9.490	-9.470	-8.621	-8.563	-8.483	-8.372	-8.311
Energy gap (eV)	3.610	3.299	2.403	2.453	2.473	2.575	2.514
Ionisation potential (eV)	9.490	9.470	8.621	8.563	8.483	8.372	8.311
Electroaffinity (eV)	5.880	6.172	6.219	6.111	6.010	5.798	5.797
Electronegativity (eV)	7.685	7.821	7.420	7.337	7.246	7.085	7.054
Chemical hardness (eV)	1.805	1.649	1.201	1.226	1.237	1.287	1.257
Chemical softness (eV)	0.277	0.303	0.416	0.408	0.404	0.388	0.398
Electrophilicity index (eV)	16.363	18.544	22.916	21.947	21.233	19.498	19.797

**Table 5:** The O-H bond dissociation enthalpies (BDE), ionization potentials (IP), proton dissociation enthalpies (PDE), proton affinities (PA), electron transfer enthalpies (ETE) in kJ/mol obtained at B3LYP/6-311++G** level of theory.

Parameter	3-Hydroksyflavone	3,7-Dihydroksyflavone	Galangin	Kaempferol	Quercetin
**BDE**					
**Gas phase**	356.05	354.80	349.07	343.97	344.42
**Water**	338.02	332.52	329.25	323.07	322.74
**Methanol**	345.17	343.17	338.90	332.72	332.43
**IP**					
**Gas phase**	745.31	725.87	726.74	703.97	525.83
**Water**	477.43	472.30	465.83	447.35	444.31
**Methanol**	496.65	494.88	487.59	471.45	463.38
**PDE**					
**Gas phase**	926.63	944.82	938.22	955.90	1134.49
**Water**	-17.66	-18.04	-14.83	-2.52	0.19
**Methanol**	32.26	32.04	35.06	45.02	52.80
**PA**					
**Gas phase**	1413.82	1417.13	1237.98	1399.81	1397.58
**Water**	129.89	128.57	118.96	123.74	123.55
**Methanol**	183.97	186.30	175.23	180.05	179.89
**ETE**					
**Gas phase**	258.12	253.57	426.98	260.06	262.74
**Water**	333.02	328.85	335.18	324.23	324.09
**Methanol**	348.09	343.76	350.56	339.57	339.43

Most parameters show a linear dependence on the number of OH groups. BDE and IP decrease in the series 3-hydroksyflavone → 3,7-dihydroksyflavone → galangin → kaempferol → quercetin. PDE increases in the series, while PA and ETE show only slight differences.

### Experimental ^1^H and ^13^C NMR, IR, Raman study

**Fig 4:**
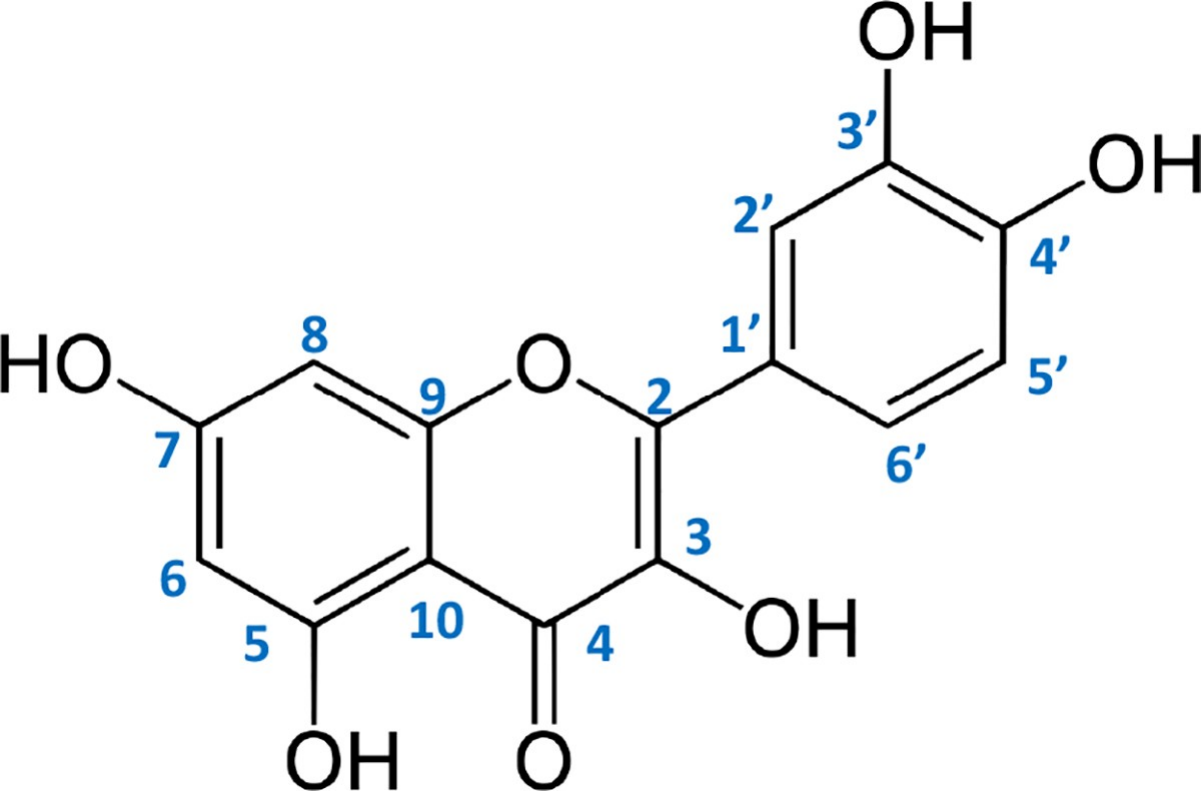
Atom numbering in flavone molecule.

The ^1^H and ^13^C NMR spectra describe the density and the electronic charge distribution which determines the reactivity and the biological activity of molecules. In Table 6, the chemical shifts from the NMR spectra are shown. Atom numbering is depicted in Fig 4. The assignments of the bands from the FT-IR and FT-Raman spectra of studied ligands (Table 7, S1 and S2 Figs of S1 File) were done based on the literature data [24] and calculations at the B3LYP/6-311++G** level.

**Table 6:** The chemical shifts δ [ppm] from the experimental and theoretical ^1^H and ^13^C NMR spectra of studied ligands.

Ring	Atom no.	Chromone		Flavone		3-Hydroxyflavone		3,7-Dihydroxyflavone		Galangin		Kaempferol		Quercetin		
		Exp.	Calc.	Exp.	Calc.	Exp.	Calc.	Exp.	Calc.	Exp.	Calc.	Exp.	Calc.	Exp.	Calc.	
	**C4**	176.35	181.52	177.08	182.07	173.00	177.34	172.36	175.33	176.3	175.00	175.91	175.17	175.90	178.09	**C4**
	**C9**	155.95	163.13	162.55	163.14	154.56	161.68	162.57	163.29	164.24	164.98	163.89	164.74	163.94	164.86	**C9**
**C**	**C2**	156.90	162.59	155.67	171.17	145.12	149.53	156.58	148.98	160.77	146.89	160.71	147.28	160.78	150.109	**C2**
	**C3**	112.26	117.83	134.27	112.01	139.11	145.77	144.15	144.05	156.43	144.83	159.19	143.74	156.20	142.80	**C3**
	**C10**	124.91	30.28	118.51	129.58	121.30	124.90	114.28	119.18	98.34	108.89	98.20	108.58	98.25	108.85	**C10**
	**H3/OH3**		6.33		6.89		7.32		7.31		7.59		7.26		7.38	
	**C1’**	-	-	131.13	137.99	131.31	137.00	131.50	137.28	137.12	137.11	146.81	128.83	146.85	128.39	**C1’**
	**C4’**	-	-	129.09	137.27	129.83	135.45	129.54	134.94	130.96	135.30	135.66	164.42	145.11	153.35	**C4’**
**B**	**C3’**	-	-	126.33	133.87	128.48	133.70	128.49	133.93	129.96	133.66	129.51	118.59	135.80	148.76	**C3’**
	**C5’**	-	-	126.33	134.07	128.48	133.43	128.49	133.68	129.34	133.32	129.51	118.60	135.80	119.16	**C5’**
	**C2’**	-	-	125.49	131.69	127.65	130.27	127.39	130.44	128.54	130.14	121.67	132.38	120.05	114.59	**C2’**
	**C6’**			125.49	132.27	127.65	135.02	127.39	134.44	127.56	134.21	121.67	136.39	115.67	129.54	**C6’**
	**H2’**			8.46	8.37	8.2	8.60	8.17	8.54	8.15	8.44	8.14	8.36	7.57	7.76	
	**H3’/OH3’**			7.52	7.70	7.53	7.80	7.52	7.82	7.52	7.76	6.92	7.18	6.89	4.65	
	**H4’/OH4’**				7.74		7.67		7.64		7.62		4.95		5.69	
	**H5’**				7.76		7.76		7.69		7.68		9.98		7.17	
	**H6’**			8.46	8.18	8.2	9.08	8.17	9.08	8.15	8.98	8.15	8.83	7.68	8.38	
	**C9**	155.95	163.13	162.55	163.14	154.56	161.68	162.57	163.29	164.24	164.98	163.89	164.74	163.94	162.45	**C9**
	**C7**	134.16	139.01	131.79	139.35	133.64	139.76	138.48	168.23	145.75	168.00	156.18	167.76	147.76	169.52	**C7**
**A**	**C5**	124.23	130.66	124.77	129.69	127.79	129.91	126.60	131.68	114.05	164.73	115.44	164.60	115.13	168.06	**C5**
	**C6**	125.42	130.28	123.32	130.28	124.49	129.95	114.94	117.37	103.24	102.11	103.05	101.84	103.08	101.61	**C6**
	**C10**	124.91	130.28	118.51	129.58	121.30	124.90	114.28	119.18	98.34	108.89	98.20	108.58	98.25	106.61	**C10**
	**C8**	118.42	123.45	106.94	122.83	118.36	122.98	102.01	106.23	93.59	96.64	93.48	98.63	93.42	96.69	**C8**
	**H5/OH5**		8.47		8.36		8.42		8.29		5.29		5.23		5.24	
	**H6**		7.58	7.88	7.67	7.42	7.69	6.93	7.00	6.21	6.22	6.19	6.19	6.19	6.18	
	**H7/OH7**		7.87		7.95		7.98		5.01		4.90		4.86		4.86	
	**H8**		7.72	7.78	7.82	7.69	7.87	7.45	7.18	7.08	6.66	6.44	6.61	6.41	6.57	

**Table 7:** Wavenumbers and intensity of selected bands from the experimental IR and Raman spectra of studied compounds.

Chromone		Flavone		3-hydroxyflavone		3,7-Dihydroxyflavone		Galangin		Kaemferol		Quercetin		Assignment
IR	R	IR	R	IR	R	IR	R	IR	R	IR	R	IR	R	
				3208 s		3575–3259 s		3500–3184 s		3352–3325 s		3390–3250 s		νOH
3085 m*	3070 vs	3059 w	3068 m	3072 w	3072 w		3078 w		3070 m	3079 w	3075 w		3080 w	νCH
1650 vs	1669 m	1646 vs	1634 vs	1607 vs	1618 vs	1610 vs	1637 vs	1658 vs	1661 s	1657 vs	1649 w	1664 s	1661 w	νC = O
1616 s	1636 vw	1619 m	1618 m	1627 sh		1626 s		1632 vs	1628 vs	1619 vs	1608 vs	1612 vs	1608 vs	νCC
1600 m	1615 s	1606 m	1604 s		1596 s		1599 s	1607 vs	1603 vs	1599 vs				νCC
1566 m	1567 m	1569 m	1570 m	1562 s	1567 s	1572 s	1576 m	1565 s	1567 s	1564 m	1558 s	1562 s	1548 s	νCC
						1520 m	1521 vw	1514 m	1522 w	1511 s	1520 m	1522 s		νCC
1474 sh		1496 m		1481 s		1492 w	1499 w		1496 w					νCC
1462 vs	1463 w	1466 s	1470 w	1470 s	1472 w	1467 s	1450 m	1468 w	1453 w		1443 m	1462 m	1440 s	νCC
1405 s	1506 w	1406 w		1416 s	1414 m	1428 m	1416 m		1399 w		1422 m	1408 m	1403 m	νCC
				1286 s	1310 m	1284 vs	1310 m	1319 s	1301 m	1319 s	1319 s	1320 s	1326 s	βOH
1235 m	1241 m	1225 m	1235 m	1212 s	1230 w	1208 m		1219 s	121 9 w	1219 s	1215 w	1200 s	1220 w	νCOC, νCC
				1184 m	1189 w	1177 vs	1177 m	1175 vs	1187 w	1166 vs	1171 s	1168 vs	1177 w	νC-OH
1126 s	1128 m	1129 s	1132 w	1130 s	1132 w	1133 s	1140 w	1126 m		1112 m	1119 w	1132 m	1138 w	βCH
1078 w	10 86 w	1078 w	1080 w	1076 m		1074 w		1072 m	1089 w	1086 m	1088 w	1092 m	1113 m	νCH
1035 m	1036 m	1044 m	1046 w	1034 m	1035 w	1032 w		1031 m						νCH
1011 m	1014 m	1010 w	1000 m	999 m	993 m	1008 w	1001 s	1008 m	1002 m	1007 s	1007 w	1014 s	1014 vw	νCH
969 m	966 w	959 w	963 vw	956 w	956 w	964 w	965 w	977 m	978 m	977 m	979 w			νCH
		906 m	907 vw	898 m		884 m		882 m		882 s	882 w			
841 s		851 m		853 w	839 w	826 m	844 w	836 m		828 m	819 w	824 s		γCH
775 s	774 w	769 s	774 w	776 s	777 w	772 m	767 w		795 vw			796 m	786 w	
758 s		756 s		759 vs		759 m		769 m	769 vw	768 w		785 w	771 w	γCH
680 m		687 m	674 w	704 s	707 w	691 s	699 w	702 m	709 w	704 m	729 w	702 m	721 w	αCCC
	686 w	673 m		690 s	674 w			690 sh	688 vw	687 w	688 w	681 s	661 w	αCCC
		648 w		657 w		656 vw		636 m	639 w	638 m	639 w	659 w		γC = O
				623 m	621 w	623 m	621 w	617 w	614 w	616 w		639 m	640 w	γOH
	576 m		578 w		579 w		585 w		580 w		584 w		604 m	αCCC
525 m	534 w	528 m	513 w	510 w	513 vw	512 w	517 w	526 w	525 w	521 w	522 w	527 w	522 w	αCCC
470 m		500 w		474 m		473 m		470 w		463 w		450 w		ϕCC
404 m		456 w		438 w				412 w		410 w		408 w		ϕCC

symbols denote: ν—stretching, β—deforming in-plane, γ—out of plane bending vibrations; α—deforming in plane of the aromatic ring; ϕ–deforming out of plane of the aromatic ring s—strong, m—medium, w—weak, v—very, sh—shoulder.

### Statistical analysis

**Fig 5:**
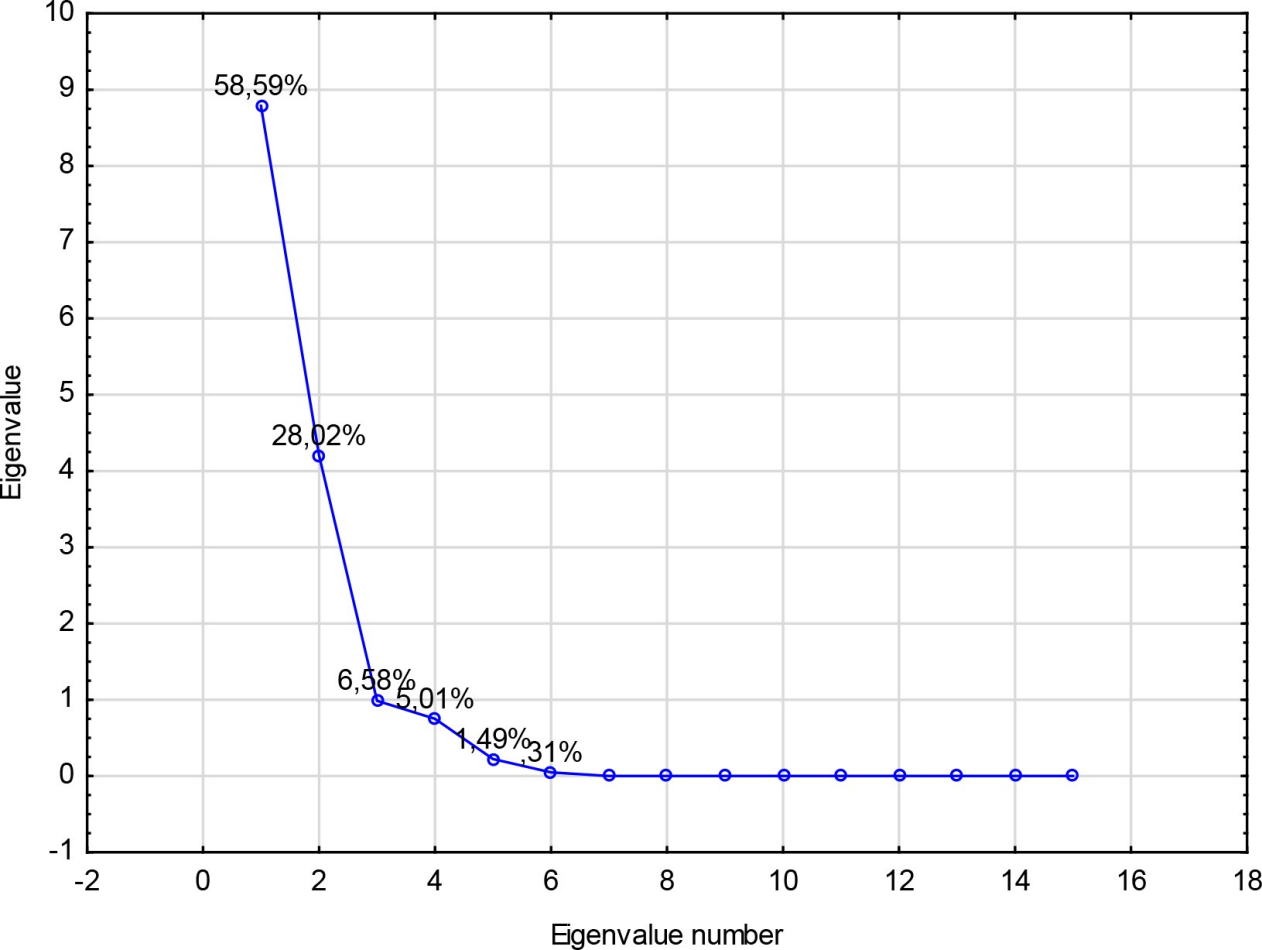
Scree plot and percentage of total variance accounted for by each factor for principal component analysis.

**Fig 6:**
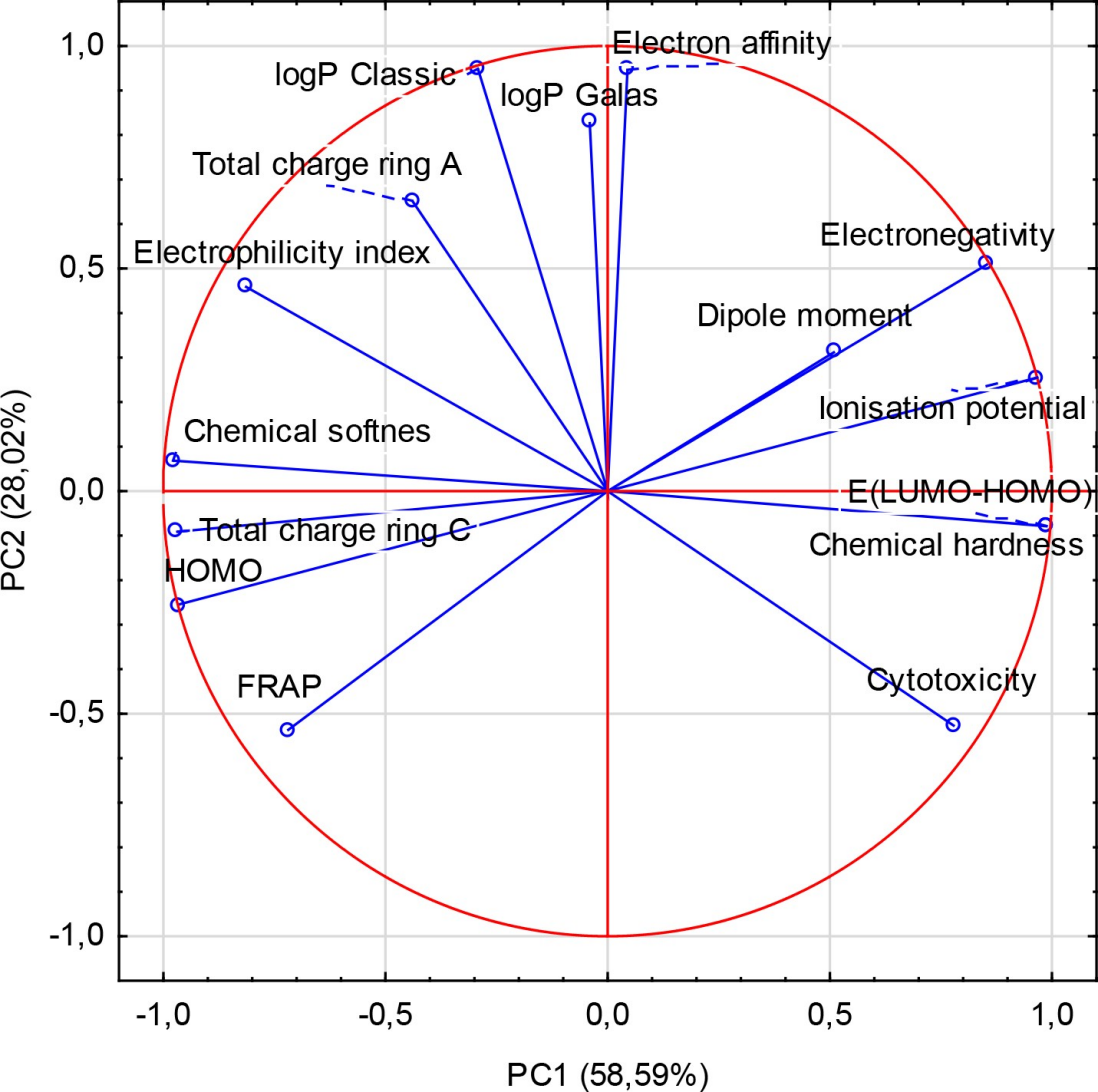
Principle component analysis of biological properties (i.e. FRAP values, IC_50_ for the cytotoxic activity towards Caco-2 cell line) and calculated physicochemical parameters.

The principal component analysis (PCA) was done to identify the correlation between the different variables. All descriptors obtained for the series of 7 ligands were submitted to principal components analysis, and two components were obtained (Fig 5). The first two PC1, PC2 contributed 58.59%, 28.02% respectively, to the total variance, and the total information was estimated to be 86.61%. The Pearson correlation coefficients are summarized in Table 8, and the descriptors are represented in a correlation circle in Fig 6.

**Table 8:** The correlation matrix between different obtained descriptors.

	FRAP	logP Classic	logP Galas	Cytotoxicity	HOMO	E(LUMO-HOMO)	Total charge ring A	Total charge ring C	Ionisation potential	Dipole moment	Electron affinity	Electron negativity	Chemical hardness	Chemical softness	Electrophilicity index
FRAP	1,000														
logP Classic	-0,339	1,000													
logP Galas	-0,265	0,747	1,000												
Cytotoxicity	-0,388	**-0,710**	-0,596	1,000											
HOMO	**0,829**	0,042	-0,179	-0,617	1,000										
E(LUMO-HOMO)	-0,620	-0,376	-0,053	**0,775**	-0,937	1,000									
Total charge ring A	0,143	0,694	0,643	**-0,790**	0,250	-0,419	1,000								
Total charge ring C	**0,789**	0,202	-0,080	**-0,716**	0,961	-0,949	0,378	1,000							
Ionisation potential	**-0,829**	-0,042	0,179	0,617	-1,000	0,937	-0,250	**-0,961**	1,000						
Dipole moment	-0,372	0,158	0,096	0,207	-0,570	0,490	0,073	-0,340	0,570	1,000					
Electron affinity	-0,666	**0,915**	0,659	-0,370	-0,279	-0,073	0,441	-0,136	0,279	0,282	1,000				
Electronenegativity	**-0,925**	0,242	0,356	0,424	-0,956	0,794	-0,084	**-0,879**	0,956	0,583	0,548	1,000			
Chemical hardness	-0,620	-0,376	-0,053	**0,775**	-0,937	1,000	-0,420	**-0,949**	0,937	0,490	-0,073	0,794	1,000		
Chemical softnes	0,597	0,368	0,018	**-0,740**	0,931	-0,998	0,384	**0,941**	-0,931	-0,486	0,083	-0,786	-0,998	1,000	
Electrophilicity index	0,207	0,706	0,274	**-0,772**	0,677	-0,888	0,498	**0,743**	-0,677	-0,304	0,511	-0,434	-0,888	0,898	1,000

## Discussion

Both DPPH and FRAP assays revealed that the distinct rise of the antioxidant activity of tested ligands depends on the number of hydroxyl substituents in the ring, i.e. in the series: chromone → flavone → 3-hydroxyflavone → 3,7-dihydroxyflavone → galangin → kaempferol → quercetin. Our experimental data are generally in accordance with the literature survey. The literature data showed that flavone has the lowest IC_50_ value—over 450 μM in DPPH tests [25]. In analogous DPPH radical reduction studies, IC_50_ values were found to be 385±19 μM for 3-hydroxyflavone and 65 μM for 3,7-dihydroxyflavone respectively [26,27] Galangin, kaempferol and quercetin are characterized by a noticeable increase in antioxidant properties described by values of EC_50_ = 13.91 μM [28] and IC_50_ = 11 μM for galangin, IC50 = 41.2 μM [29], 7.1 μM [28] and 47.93±0.01 [30] μM for kaempferol. Quercetin proved to be the most effective antioxidant with the lowest IC_50_ and EC_50_ values in DPPH tests (IC_50_ = 16.2±1.1 μM [31], 8.9 μM [29], 6.7±0.17 μM [27] and EC_50_ = 5.5μM [32], 39±0.1μM [33], 112.5±3.3 μM [34], 2.79 μM [28].

While comparing the results obtained by different authors it should be kept in mind that the experimental conditions such as the concentration of DPPH, pH of the solution, temperature, presence of metal ions strongly affect the obtained IC_50_/EC_50_ parameters. The results obtained in the same experimental conditions allow relating the antioxidant activities in the series more precisely (Fig 2).

The antioxidant activity of phenolic compounds is related to the ring structure of the molecule and the presence of the hydroxyl substituents mainly in the B ring. The number of hydroxyl groups is positively correlated with the antioxidant potential of the studied compounds. It is assumed that the antiradical action of flavonoids rely on their direct reaction with radical and formation of radical from catechol moiety. However, Musialik et al.’s results in methanol support their conclusion that in ionizing solvents the initial fast DPPH• + QH2 (quercetin) reaction involves the QH–anion [35]. In such solvents, the reaction in quercetin and other phenols occurs by the SPLET mechanism. Decreasing electron transfer energies together with the decrease of bond dissociation energies for ligands from the end of the series (Table 6) well explain the phenomenon of their pronounced antioxidant activity.

**Fig 7:**
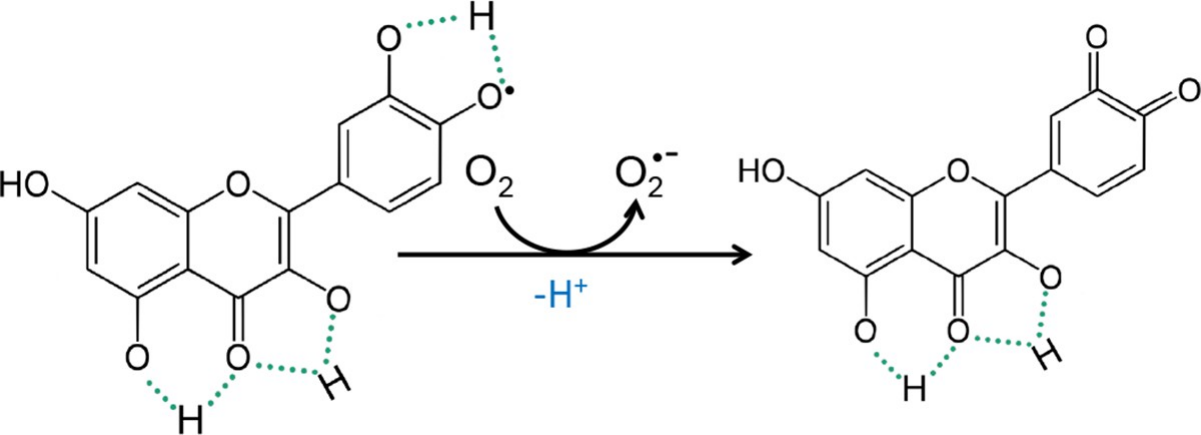
The mechanism of pro-oxidative activity [36].

However, the effectiveness of ROS scavenging by phytochemicals is closely related to their concentration and in high doses, prooxidative effects are observed—generation of ROS in the processes of autooxidation, redox-cycling. The presence of a different number of hydroxyl groups in the B ring of flavonols may determine their antioxidant activity but also plays an important role in their toxicity and biological activity. Current studies indicate questionable stability of phenoxyl radical, which results in pro-oxidative reactions. It is believed that the pro-oxidative activity of these compounds is directly proportional to the total number of hydroxyl groups. The flavonoid phenoxyl radical can interact with oxygen resulting in the formation of quinones and superoxide anions. This reaction may take place in the presence of a higher concentration of transition metal ions and may be responsible for the undesirable flavonoid pro-oxidation effect (Fig 7). In general, low BDE and IP values indicate a high antioxidant activity, but an extremely low IP value may result in a change from an antioxidant to a prooxidant character. Phenolic compounds with a small IP value tend to act as prooxidants in the presence of reactive oxygen species and increase the cytotoxic potential of phenolics.

Quantum chemical calculations provide some insight into the molecular mechanisms of radical scavenging and anti-inflammatory activity of the investigated phenolic compounds. The values of the energy of HOMO orbitals are important information on the mechanism of antioxidant action. Generally, the higher HOMO orbital energy of molecule the better its electron-donating properties. The lower ionization potential (IP = —HOMO) the lower the energy to remove an electron. In the series chromone → flavone → 3-hydroxyflavone → 3,7-dihydroxyflavone → galangin → kaempferol → quercetin the energy of HOMO orbitals increases and IP decreases (Fig 3, Table 5). This suggests that the antioxidant activity of the molecules rises in the same order as proven in the DPPH and FRAP assays. Despite the HOMO and LUMO orbitals are similarly distributed overall the molecules, it can be noticed that HOMO orbitals are localized on the hydroxyl groups at C3 and C3’position. This means that the hydroxyl groups in the B and C rings can be easier attacked by free radical than the other ones. The difference between the energy of HOMO and LUMO orbitals (ΔE) describes the stability and reactivity of chemical compounds. Molecules with a large ΔE are characterized as stable and low reactive. On the other hand, small ΔE means low stability and higher molecular reactivity. These values dramatically decrease for the following hydroxyl derivatives of chromone. Substitution of the OH group to the skeleton of flavone rises the reactivity of the molecules. For instance, the experiment shows that the anti-DPPH activity of quercetin with a catechol-like substitution on the B ring is about 9 times greater than that of galangin that does not possess the two hydroxyl groups in the B ring (Fig 2) and more than 50 times greater than that of 3-hydroxyflavone.

Generally, three pathways are assumed for the antioxidant mechanism of phenolic molecules [37]. The hydrogen atom transfer (HAT) mechanism in which the free radical removes one hydrogen atom from the antioxidant and the antioxidant becomes a radical. BDE (bond dissociation enthalpy) is used to estimate the reactivity of molecule in HAT mechanism (Table 9). The second mechanism, single-electron transfer followed by proton transfer (SET-PT). It is a two-step mechanism, (a) first the antioxidant reacts with the free radical to form a cation radical of antioxidant and an anionic form of the radical (an IP, ionization potential, is related with this mechanism), then (b) the cation radical of antioxidant decomposes to a radical and a proton (PDE, proton dissociation enthalpy parameter describes the reaction). The third mechanism, a sequential proton loss electron transfer (SPLET). It is as well a two-step mechanism. First phenolic antioxidant dissociates into an anionic form and a proton (PA, proton affinity is related with the mechanism). Second, the anion reacts with the free radical and a radical form of the antioxidant and a neutral molecule occurs (ETE, electron transfer enthalpy reflects the reaction).

**Table 9:** Different mechanisms of antioxidant action and the parameters describing the activity [37].

Mechanism	Reaction	Parameter
HAT	(1) ArOH + X^•^ → ArO^•^+ XH	(1) B.D.E = H(ArO^•^) + H(H^•^)–H(ArOH)
SET-PT	(1) ArOH + X^•^ → ArOH^•+^ + X^–^	(1) IP = H(ArOH^•+^) + H(H^+^)—H(ArOH)
	(2) ArOH^•+^ → ArO^•^ + H^+^	(2) PDE = H(ArO^•^) + H(H^+^)–H(ArOH^•+^)
SPLET	(1) ArOH → ArO^−^+ H^+^	(1) PA = H(ArO^-^) + H(H^+^)–H(ArOH)
	(2) ArO^−^+ X^•^ + H^+^ → ArO^•^ + XH	(2) ETE = H(ArO^•^) + H(e-)–H(ArO^-^)

The bond dissociation energy (BDE) is an enthalpy change of the homolytic bond cleavage. It determines the likelihood of the HAT mechanism. The weaker the O-H bond, the lower is the BDE value and the antioxidant properties of the molecule are higher. The obtained results suggest that with the increasing number of hydroxyl substituents the antioxidant activity in HAT mechanism increases as well (Table 6).

The SET-PT mechanism is related to the IP (ionization potential) and PDE (proton dissociation enthalpy) parameters. The first one describes the first step of SET-PT mechanism, dependent on the donating ability of compounds, which is related to the electronic charge distribution over the molecule. The higher the degree of π-electron delocalization the more active the molecule. According to the IP values the ability to donate an electron increases in the series: 3-hydroxyflavone→3,7-dihydroxyflavone→galangin→kaempferol→quercetin. Moreover, an electron is donated more easily in polar media than in the gas phase. The second step of SET-PT mechanism, i.e. decomposition of a cation radical of antioxidant to a radical and a proton, occurs easier in the polar environment than in the gas phase. The lowest values of PDEs are for 3,7-dihydroxyflavone (in water and methanol) and 3-hydroxyflavone in the gas phase. The cation radical of quercetin is the most stable. Because the first step is the most important from the thermodynamic point of view, therefore the antioxidant activity of compounds according to the values of IPs can be ordered as follows: 3-hydroxyflavone→3,7-dihydroxyflavone→galangin→kaempferol→quercetin (Table 6).

The SPLET mechanism includes two steps. The first step is the process of anion formation, which according to the obtained values of proton affinities (PA), is easier in the solution than in the gas phase. The high average difference between the PA calculated for gas and solution (~1200 kJ/mol) is mainly caused by the high solvation enthalpies of proton and anion (Table 6). The proton is most easily cleaved from the galangin, then from quercetin and kaempferol. It seems that the formation of -O^-^ anions by C3-OH and C7-OH hydroxyl groups is easier for galangin than for other compounds in the series. The second step of SPLET mechanism is governed by electron transfer enthalpies (ETE), which generally are lower for isolated molecule (i.e. in the gas phase) than in the solution. This indicates that the gas phase facilitates the formation of the radicals of chromone derivatives. In the gas phase, the lowest ETE is for 3,7-dihydroxyflavone whereas in water and methanol for quercetin and kaempferol. The highest ETE parameters are for galangin both in the gas phase and in the polar solvent. It means that for galangin the process of radical formation is the hardest. The antioxidant activity of the molecules in the SPLET mechanism, according to the PAs, can be ordered as follows: 3-hydroxyflavone→3,7-dihydroxyflavone →kaempferol→quercetin→galangin.

**Fig 8:**
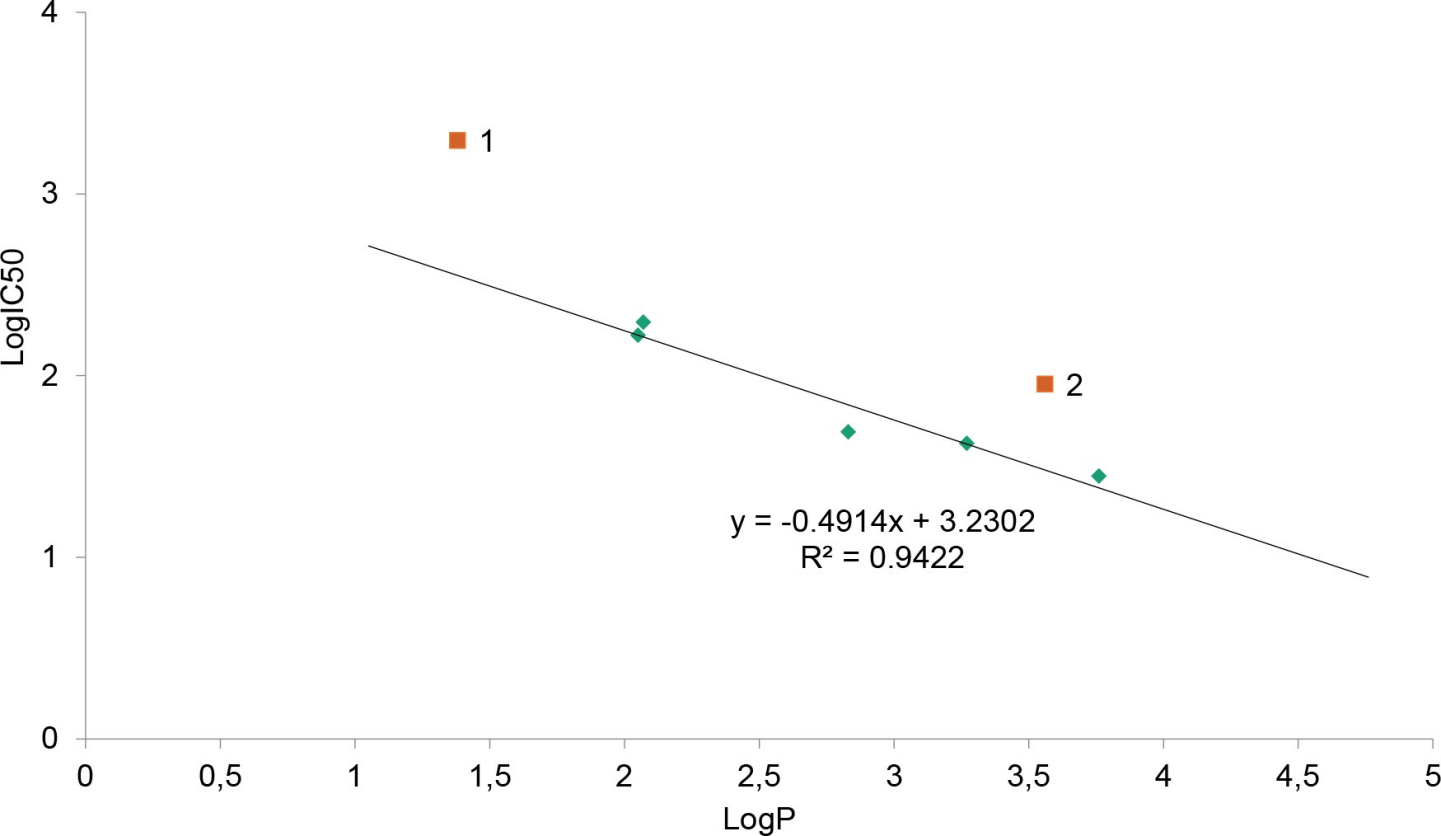
The toxicity on lipophilicity (ACD/LogP Classic) dependence of the described series of compounds. The two exceptions are chromone (1) and flavone (2).

Another biological activity of the series of 7 ligands discussed in the paper are cytotoxicity toward human epithelial colorectal adenocarcinoma Caco-2 cells (expressed as IC_50_ [μM]) and lipophilicity (as LogP) (Tables 1 and 2). LogP is used in the pharmaceutical industry to understand the behaviour of drug molecules in the body. This is because lipophilicity is a major determining factor in a compound’s absorption, distribution, penetration across vital membranes and biological barriers, metabolism and excretion. If an adequate concentration of a drug in the target tissue cannot be reached or maintained, even the most potent in-vitro substance cannot be an effective drug. The highly water-soluble substances will easily reach hydrophilic compartments of the tissue, but at the same time may be rapidly excreted. In turn, lipophilic compounds may be sequestered by fatty tissue and therefore difficult to excrete. This may lead to an accumulation that will impact the systemic toxicity of the substance. Depending on the administration route of a given compound and its target milieu in the biological environment, an ideal candidate for a drug must have lipophilicity allowing for penetration through relevant barriers. Thus, for example, a drug targeting the central nervous system should ideally have a logP value around 2 [38] while for oral and intestinal absorption the ideal value is 1.35–1.8 [39]. Therefore, LogP helps to predict the likely transport of a compound around the body. It also affects formulation, dosing, drug clearance, and toxicity. Though it is not the only determining factor, it plays a critical role in helping scientists limit the liabilities of new drug candidates. Consequently to the above, the dependence of drug toxicity in a series of compounds of varied lipophilicity tends to have its optimum for a certain LogP value. As an example, such an optimum was found in the homologous series of gold-containing phosphine derivatives [40]. The hydrophilic nature of these compounds can be varied over a very large range without losing aromatic character by replacement of the phenyl substituents on the diphosphine linkages with pyridyl ligands. When the lipophilicity was related to anticancer activity it turned out, that the logarithmic free drug IC_50_ values for the CH-1 mouse lymphoma cell line bore a parabolic dependence on drug lipophilicity. Logarithmic free drug IC_50_ values and uptake rates were linearly related to lipophilicity. Host toxicity in vivo in MC-38 xenografts varied according to lipophilicity with the most selective compound having an intermediate value [40]. Similarly, in the herein described series of compounds, there can be observed a trend in toxicity related to the lipophilicity values (Fig 8, Tables 1 and 2). In general, the LogIC_50_ of the described series of compounds tends to be linearly dependent on their LogP value. There are, however, two exceptions. Chromone, the only one from the series not possessing the additional phenyl ring in the ortho- position to pyran ring oxygen (the B-ring, a characteristic of flavonoids), characterizes with the highest water solubility and the lowest toxicity (two-three orders of magnitude lower than the other compounds in the series). Also flavone, the only one of the flavonoids in the series not possessing any OH groups, displays lower toxicity (higher IC_50_) in relation to lipophilicity as could be expected in the series. Summarizing, the toxicity of the–OH group-bearing compounds in the series linearly depends on their lipophilicity. The higher lipophilicity, the higher toxicity (lower IC_50_). A general trend can be seen, that with the increase of the–OH groups number lipophilicity decreases (as can be expected) and as well decreases toxicity, along with the increasing antioxidant potential (by FRAP values and EC_50_ parameters obtained in the DPPH assay). Yet in the absence of–OH groups, the toxicity of the analogues also decreases. This interesting finding emerges a separate group of the compounds in the series, possessing the characteristic flavone backbone and at the same time at least one hydroxyl group.

In the literature, many results are describing the cytotoxic activity of the herein studied PPs in relation to different cancer cell lines (S11 Table of S1 File). Such activity is determined by the molecular structure of polyphenols, the physicochemical properties of molecules which, among other things, allows: (a) direct binding to the receptor proteins and inhibition of growth factors, tyrosine tyrosine kinases [41], (b) participation in cell signalling pathways, ROS-dependent pathways, including the antioxidant defence-related, inhibition of enzymes involved in the free radical generation [12, 42, 43] (c) scavenging of free radicals and binding (complexation) of transition metal ions (Cu^2+^, Fe^2+^), (d) modulating the activity of epigenetic factors, e.g. activation of SIRT (sirtuin) by interacting with particular amino acid residues [44], regulation of genes involved in chromatin remodelling [45], (e) generation of reactive oxygen species (pro-oxidative activity), which causes cell cycle arrest, induction of apoptosis and DNA fragmentation [12].

The molecular structure of the studied ligands was discussed in terms of calculated in B3LYP/6-311++G** level geometrical parameters, NBO atomic charges as well as experimental ^1^H, ^13^C NMR, IR, Raman spectra. The spatial arrangement of the hydroxyl group is the main factor determining the flat structure of the molecule, its energy and dipole moment (S12 Table in S2 File). The conformer no. 1 of 3-hydroxyflavone possesses the OH group directed toward the B ring what causes bending of the molecule in relation to the plane of the A and C rings. In the case of conformer no. 2 the OH group is directed toward the carbonyl C4O4 moiety what allows the flat structure of the 3-hydroxyflavone molecule. Conformer no.1 possesses four intramolecular hydrogen bonds (i.h.b.) of medium strength (the lengths are in the range: 2.557–2.691A) and higher energy than the one no. 2 with five i.h.b. (2.057–2.633A) including two strong bonds (S1 Table of S1 File). The total atomic charge is negative in the case of A and B rings, and positive for the C ring (S2 Table of S1 File). The two conformers differ the most in the total charge of the C ring.

The conformer no. 1 of 3,7-dihydroxyflavone possesses the hydroxyl group O3H3 directed toward B ring what causes the twisting of B ring in relation to the plane of A and C rings. The twisting of the conformer results from the steric effects. In the case of conformer 2, the O3H3 group is directed in an opposite direction (i.e. toward O4 atom) and as a consequence the molecule became flat and its energy decreases comparing with the conformer no.1. The conformer no. 3 differs from the one no. 2 in the spacial arrangement of the O7H7 group which is directed toward H8 atom. It causes a decrease in the strength of the O7···H8 hydrogen bonds and a slight decrease in the energy of the molecule. The conformer no. 4 differs from the one no. 3 in the bending of the O3H3 group towards B ring what causes twisting of the B ring in relation to the plane of the A and C rings (similar situation as it was in the case of conformer no. 1). The spacial arrangement of the O3H3 group decides about (a) the structure (the conformers nos. 2 and 3 are flat whereas the nos. 1 and 4 aren’t), (b) the bond lengths and values of angles in the molecules (S3 Table of S1 File) and (c) the numbers and strength of the intramolecular hydrogen bonds. The conformers with the lowest energy—nos. 2 and 3 possess one more hydrogen bond comparing with the conformers nos. 1 and 4, i.e. between the O4 and H3 atoms which is the strongest one. According to Jeffrey [46], the hydrogen bonds can be divided due to their length: 2.2–2.5 Å (strong), 2.5–3.2 Å (medium) and 3.2–4.0 Å (weak). Having regard to the fact that calculations are burdened to certain error comparing with the experiment, the intramolecular hydrogen bonds present in the molecule of 3,7-dihydroxyflavone can be assigned as strong: O4···H3, O3···H6’ and medium O7···H8, O1···H8, O4···H5 in the case of conformers 2 and 3. Whereas in the conformers nos. 1 and 4 all hydrogen bonds are of medium strength. Therefore, it can be concluded that in the molecule of 3,7-dihydroxyflavone the number and the strength of the intramolecular hydrogen bonds determine the energy and stability of the molecule. The total NBO charge of the A, C and B rings is in the range: (-0.186e)-(-0.200e), 0.599e-0.652e and (-0.659)-(-0.663e), respectively (S4 Table of S1 File). The most distinct differences between particular conformers concern the atomic charges gathered on the atoms that take part in the formation of the intramolecular hydrogen bonds and the C ring (engages in the largest amount of the hydrogen bonds).

The eight conformers of galangin differ in the spatial arrangement of the hydroxyl groups which affects the energy and dipole moments of molecules. The energy of conformers decrease in the order: conformer no.5→no.1 = no.2 = no.3 →no.6→no.4→no.7→no.8. Conformers nos. 1–3 and 6 possess one strong intramolecular hydrogen bond C4 = O···HO, whereas conformers nos. 7 and 8 have one more hydrogen bond C4 = O···HO and are characterized by the lowest energy (conformers nos. 4 and 5 do not have this type of hydrogen bonds). Conformer 8 possesses lower energy by 0.001 a.u. and almost the twice lower value of the dipole moment comparing with conformer 7, so the conformer no. 8 may be considered as the most stable structure. The conformers nos. 1–5, which are not flat, differ much in the length of the C1’-C2 bond compared with the flat structures of conformers 6–8. These conformers vary in length of the C-C in the rings, but also the C = O and O-H bonds engaged in the hydrogen bonds, i.e. C4 = O4, O3-H3 and O5-H5 which become weaker after participation in hydrogen bond formation (S5 Table of S1 File). The total NBO atomic charge calculated for the particular rings is negative for the rings A and B and positive for the C ring (S6 Table of S1 File). The most stable conformer no. 8 possesses higher negative NBO charge gathered on the oxygen atoms of the C = O and OH groups (which take parts in the hydrogen bonds) compared with the conformer no. 5 (without the intramolecular hydrogen bonds C4 = O···HO).

The presence of the hydroxyl group in the B ring of kaempferol causes a decrease in the energy of molecule comparing with galangin molecule with no OH group in B ring. The B ring of conformer no.1 is slightly twisted in relation to the plane of A and C ring. Conformers nos. 2–5 are flat, their energy is not much lower compared with the conformer no. 1 (S7 Table of S1 File). The differences in the spatial arrangements of the OH groups in the molecules of conformers 2–5 do not affect the length of the C-OH bond and the energy of molecules, but greatly affect the values of their dipole moments. The hydrogen bond C4 = O···HO-C5 is stronger than C4 = O···HO-C3 and is more sensitive for the position changes of the rest of OH groups in the rings. The spacial arrangement of the hydroxyl substituent influences the values of the atomic charges to a lesser extent than the presence or absence of the intramolecular hydrogen bonds between O4 and H3O3 and H5O5 atoms (S8 Table of S1 File).

The conformers of quercetin nos. 1–3 possess similar values of energy. These values are lower than for the conformers no. 4–6. The conformers nos. 1–3 mostly differ from 4–6 in the presence of two hydrogen bonds between C4 = O and hydroxyl group substituted in the C3 and C5 atoms (i.e. C4 = O···HO-C5 and C4 = O···HO-C3). Breaking of the intramolecular hydrogen bonds causes lowering of molecule stability. The presence of the hydrogen bonds between C4 = O and OH strongly affects the C-C bond length in the rings of the molecule, mainly C3-C4 and C4-C10 (S9 Table of S1 File). The atomic charges on the O4, O5 and O3 atoms in the conformers 1–3 (engaged in the formation of the intramolecular hydrogen bond between the carbonyl C4 = O and hydroxyl groups C5-OH and C3-OH) are the lowest comparing the same atoms in the conformers 4–6 (partially or totally deprived of the i.h.b. between C4 = O and OH) (S10 Table of S1 File).

There are distinct differences in the experimental ^1^H, ^13^C NMR, IR and Raman spectra along the series of studied ligands. These differences are caused by the increase in the numbers of the–OH substituents in the rings. The substitution of the ring B to the skeleton of chromone causes significant changes in the electronic charge distribution of the C ring, expressed as the distinct movement of the chemical shifts assigned to the selected carbon atoms in the experimental NMR spectra of ligands (Table 6). Namely, the electronic charge distribution around the C3 and C2 atoms decreases, whereas around the C10 increases. The same conclusion can be drawn based on the values of the NBO atomic charges (S12 Table in S2 File). The substitution of the hydroxyl group in the C3 position of the C ring of flavone causes a distinct shift of the signals assigned to the C2 atom toward lower ppm values, indicating an increase in the electronic charge density around C2. The electronic charge density around the C3 atom of 3-hydroxyflavone decreases compared with flavone, which is especially visible in the value of the NBO atomic charges (Δ_NBO_ = 0.531e). The appearance of the additional OH group to the A ring, in position C7, mostly affects the density around C6 and C10 atom which increases in the molecule of 3,7-dihydroxyflavone compared with 3-hydroxyflavone. Moreover, the negative charge around C7 atom decreases (Δ_NBO_ = 0.516e). In galangin, which possesses one more OH group in the C5 position of the C ring, a decrease in the electronic charge density around the C3 atom is observed. Moreover, a distinct increase in the electronic charge density around C5, C6, C10 and C8 atoms of the A ring occurs, with an exception for a distinct decrease in the negative charge of C5 atom (Δ_NBO_ = 0.520e). In the molecule of kaempferol, the additional OH group at the C4’ atom of the B ring causes a distinct decrease in the electronic charge density around C4’ atom (Δ_NBO_ = 0.518e) and a slight increase around C2’ and C6’ atoms. Substitution of the next OH group in the B ring (i.e. quercetin) causes a decrease in the electronic charge distribution around the C3’ atom (Δ_NBO_ = 0.133e) and a slight increase around C6’ and a decrease around C2’ atom.

Every subsequent substitution of the OH group causes a decrease in the total NBO charge of particular rings by ~0.4e. Namely, the substitution of the OH group to the C3 atom of flavone reduces the total NBO charge of C ring by 0.443e. The differences between the values of total NBO charge of A ring of 3-hydroxyflavone and 3,7-dihydroxyflavone is 0.410e, whereas between 3,7-dihydroxyflavone and galangin it is 0.405e. The total NBO charge of B ring of galangin decreases after substitution of the OH group in the position of C4’ (kaempferol) (Δ = 0.439e). The substitution of another OH group in the C3’ position of the B ring (quercetin) reduces the total NBO charge by 0.400e.

In the vibrational spectra of studied ligands in the range of 1607–1664 (IR), 1618–1669 (R) cm^-1^ the very strong band assigned to the stretching vibrations of the carbonyl group C = O occurred (Table 7). The appearance of the OH group at the C3 atom caused a distinct decrease in the wavenumber of the band assigned to the C = O vibrations in the spectra of 3-hydroxyflavone [1607 (IR), 1618 (R) cm^-1^] compared with the spectra of flavone [1646 (IR), 1634 (R) cm^-1^]. This was due to the formation of the intramolecular hydrogen bonds between the C4 = O···HO-C3 and weakening of the C = O bond. In the series of 3-hydroxyflavone→3,7-dihydroxyflavone→galangin→kaempferol→quercetin, this band was shifted toward higher wavenumbers with the increase in the number of OH substituents. In the range of 1632–1405 (IR), 1636–1339 (R) cm^-1^ the bands derived from the stretching νCC vibrations occurred. These bands were sensitive to the substitution of the OH groups in the rings and generally decreased in the wavenumbers along with the above-mentioned series as the negative NBO atomic charge in the ring decreased (S12 Table in S2 File). The bands assigned to the vibrations of βOH group, i.e. ~1320 and νC-OH ~1170 cm^-1^ were respectively shifted to the higher and lower wavenumber in the series 3-hydroxyflavone→3,7-dihydroxyflavone→ galangin→kaempferol→quercetin.

The principal component analyses (PCA) and hierarchical cluster analysis were used to study the dependence between the molecular structure of the 7 ligands and their biological activity. The antioxidant activity described by the FRAP value is highly correlated (R>0.700) with the energy of HOMO orbital (R = 0.829) and the total charge of the ring C (R = 0.789), and strongly negatively correlated with the calculated ionization potential (R = -0.829) and electronegativity (R = -0.925) (Table 8). The cytotoxicity towards the Caco-2 cell line (expressed as IC_50_ [μM]) and energy gap (LUMO-HOMO) (R = 0.775) and chemical hardness (R = 0.775) are highly correlated. Moreover, cytotoxicity and logPClassic (R = -0.710), the total charge of A and C rings (R = -0.790, R = -0.716, respectively), electrophilicity (R = -0.772) are strongly negatively correlated.

**Fig 9:**
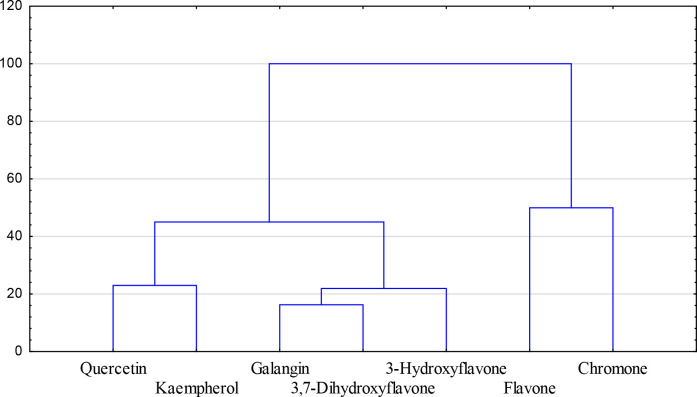
Dendrogram of the series of 7 ligands based on their biological properties (i.e. FRAP values, IC50 for the cytotoxic activity towards Caco-2 cell line) and calculated physicochemical parameters.

Taking into account all experimental and calculated data that describe the biological and physicochemical properties of the studied ligands they can be grouped into three main clusters (Fig 9). The results suggest that the number of hydroxyl substituents in the ring is a decisive factor determining the biological and physicochemical properties of the studied ligands. Namely, he first group consists of quercetin and kaempferol, which possess the hydroxyl substituent in all rings. The chromone derivatives that belong to the second group (galangin, 3,7-dihydroxyflavone, 3-hydroxyflavone) possess the hydroxyl substituent in the A and C ring, whereas the third group consists of the compounds without the–OH substituent in the ring.

## Conclusions

The appropriate selection of compounds for the research allowed a better understanding of the changes in their biological activity. The acceptor-donor properties, physicochemical parameters as well as the number and spatial arrangement of the OH groups, the numbers and strength of the intramolecular hydrogen bonds determined the distribution of the electronic charge in the chromone derivatives and therefore their stability as well as chemical and biological reactivity. Generally, the antioxidant activity of the studied ligands increased with the number of hydroxyl substituents in the ring, i.e. in the series: chromone → flavone → 3-hydroxyflavone → 3,7-dihydroxyflavone → galangin → kaempferol → quercetin. The mechanism of action of these antioxidants, which can be realized in HAT, SET-PT, SPLET pathways, strongly depends on the type of solvent. Moreover with increasing number of -OH groups the antioxidant activity in the HAT and SET-PT mechanism increases as well. The SPLET mechanism of antioxidant activity of phenols is widely described in the literature. In the first step in involves the formation of anion by the C3-OH hydroxyl group which is more favorable for galangin than quercetin. The lipophilicity, energy of HOMO orbital, energy gap (LUMO-HOMO) and the total NBO atomic charge of the A and C rings of the molecules strongly correlate with their biological activity (i.e. antioxidant or cytotoxic).

Further research on the dependency structure-activity should be continued on a larger group of structurally similar chemical compounds. Such studies provide information which is used in design biologically important molecules that can be applied in the food industry, pharmacy, medicine as antioxidant, antimicrobial agents or drugs. It will facilitate the search for more effective antioxidants or cytotoxic compounds of natural origin.

## Supporting information

S1 File (PDF)

S2 File (DOCX)
