# Supplementary material for: Correlations between molecular structure and biological activity in "logical series" of dietary chromone derivatives
Source: PLoS One. 2020 Aug 21;15(8):e0229477. doi: 10.1371/journal.pone.0229477 (PMC7444502; doi:10.1371/journal.pone.0229477)
Supplement: S1 File — (PDF) [file pone.0229477.s001.pdf]

Supporting information

Correlations between molecular structure and biological activity in "logical series" of dietary chromone derivatives

Short title: Structure and biological activity of chromone derivatives

Włodzimierz Lewandowski [1, 1], Hanna Lewandowska [2], Aleksandra Golonko [3], Grzegorz Świderski [1], Renata Świsłocka [1], Monika Kalinowska[1, 1]

1. Department of Chemistry, Biology and Biotechnology, Bialystok University of Technology, Bialystok, Poland

2. Institute of Nuclear Chemistry and Technology, Centre for Radiation Research and Technology, Warsaw, Poland

3. Department of Microbiology, Institute of Agricultural and Food Biotechnology, Warsaw, Poland;

* Corresponding authors

E-mail: w-lewando@wp.pl (WL); m.kalinowska@pb.edu.pl (MK)

Funding: This work was funded by the National Science Centre, Poland under the research project number 2015/17/B/NZ9/03581 and work number WZ/WB-IIS/5/2020 financed from funds for the education of the Ministry of Science and Higher Education.

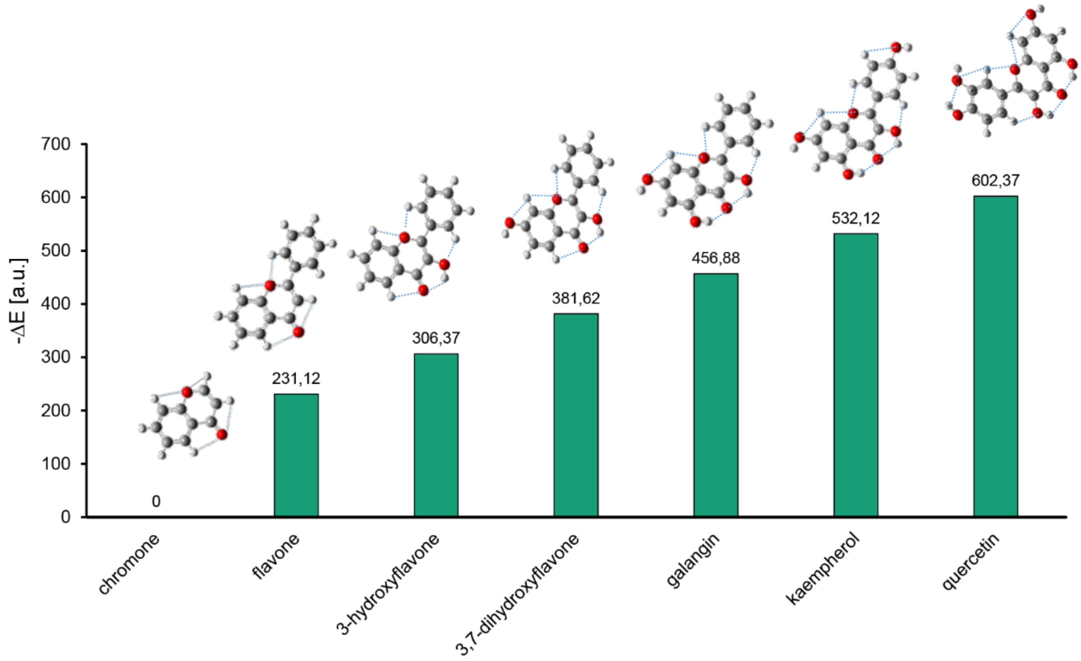

Fig S1. The energy of the most stable conformers of the studied compounds in relation to the energy of chromone.

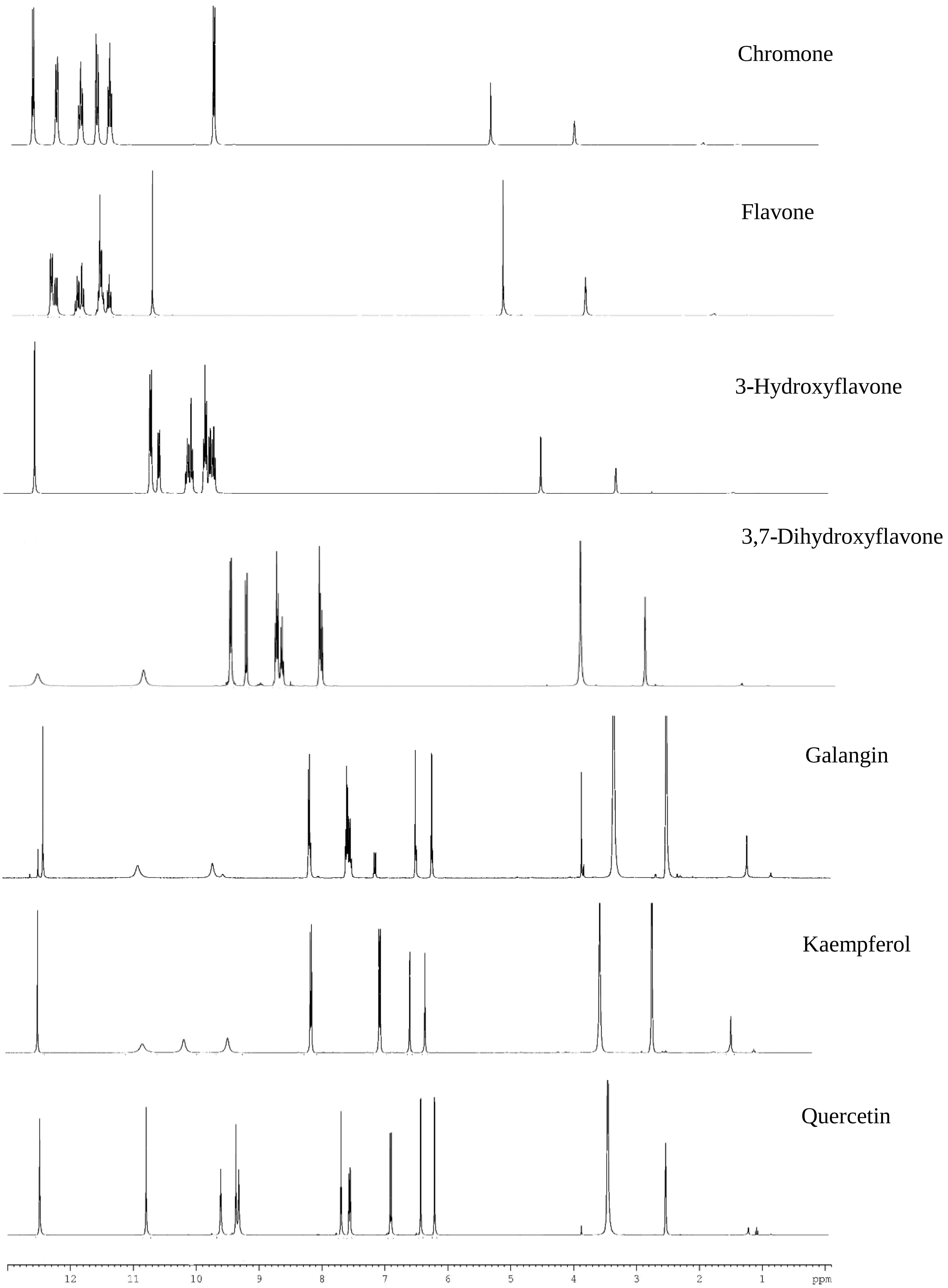

Fig S2. The ^{1}H NMR spectra of chromone derivatives.

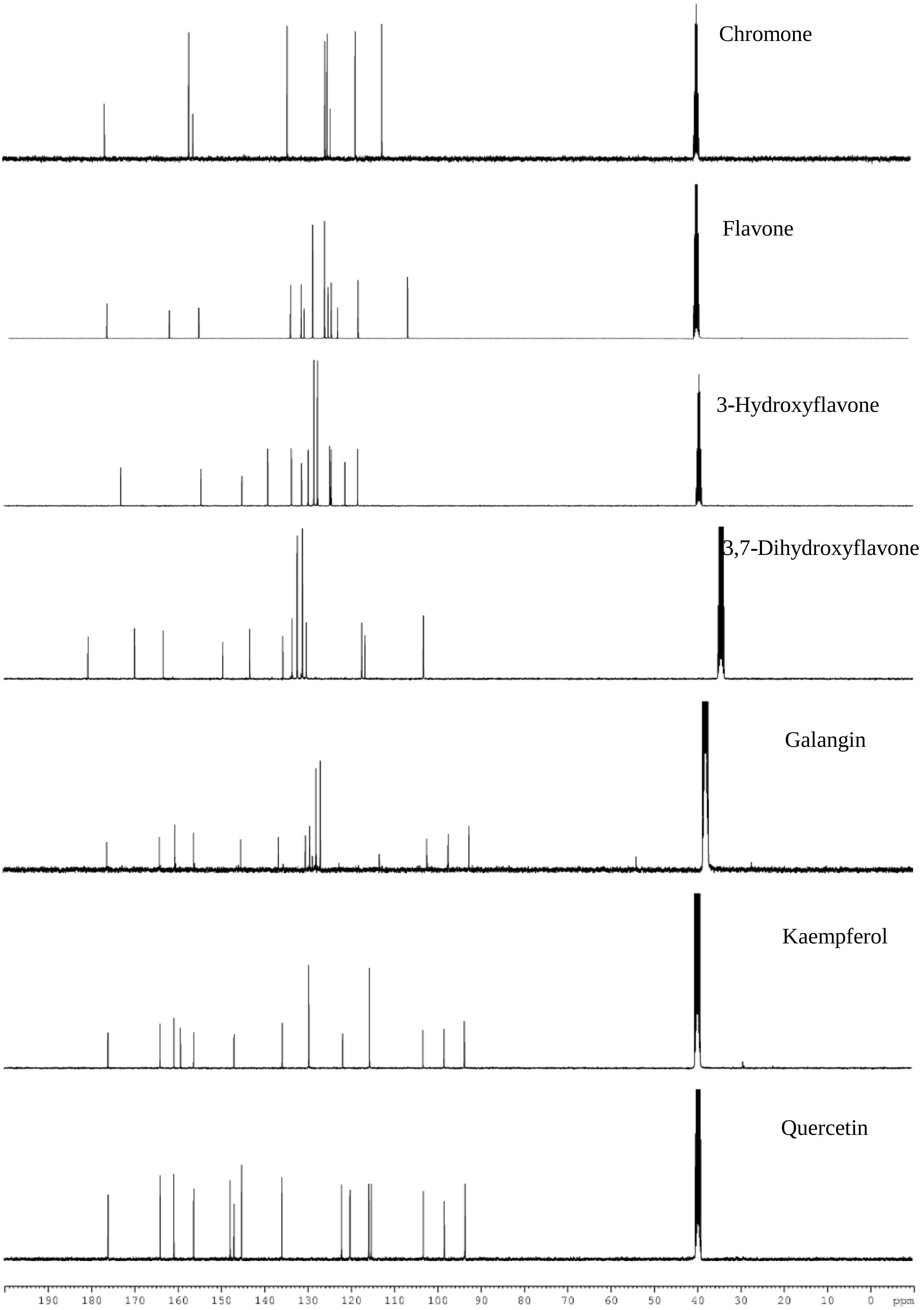

Fig S3. The ^{13}C NMR spectra of chromone derivatives.

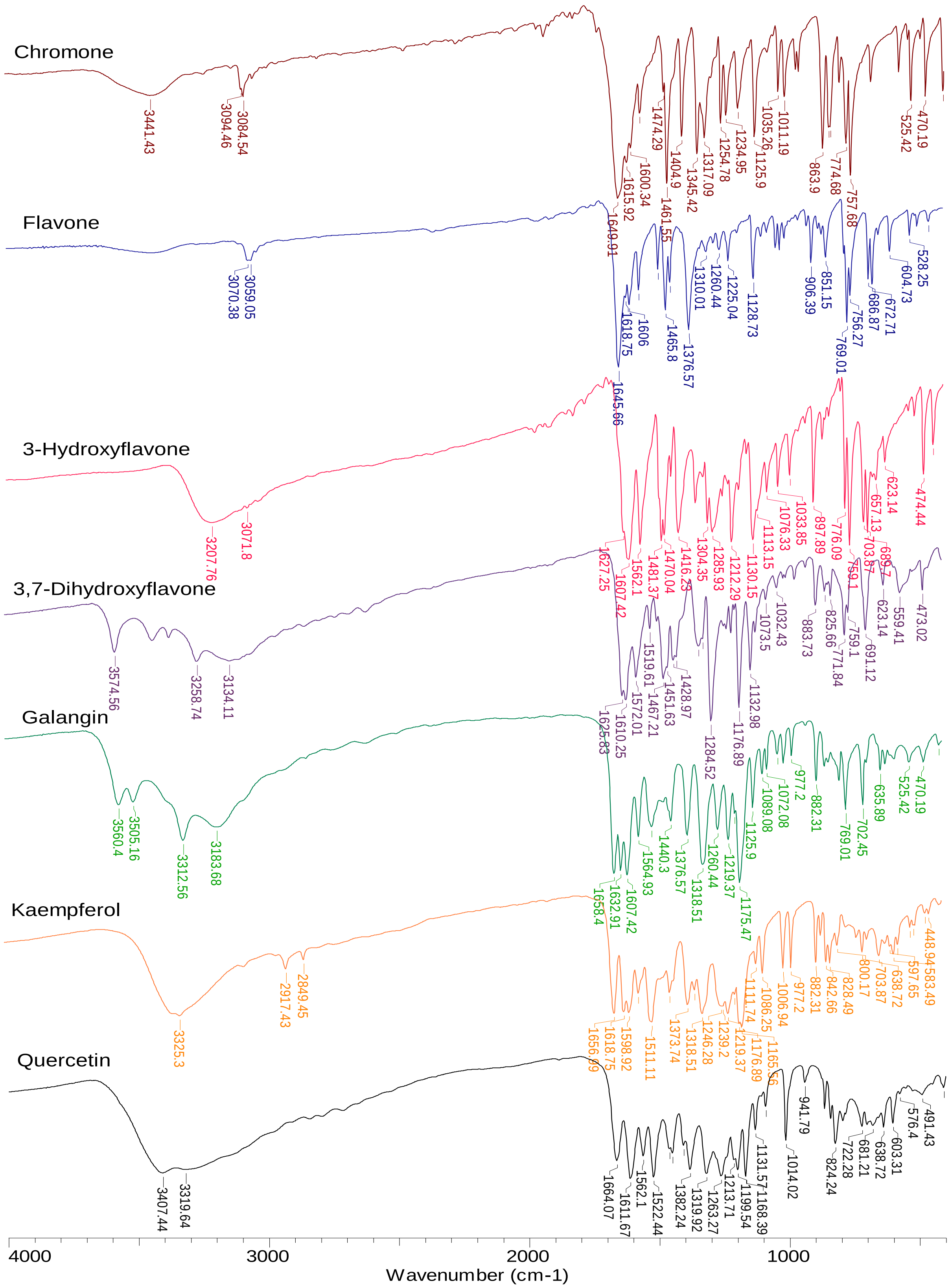

Fig S4. The IR spectra of chromone derivatives registered in the range of 400-4000 cm^{-1}.

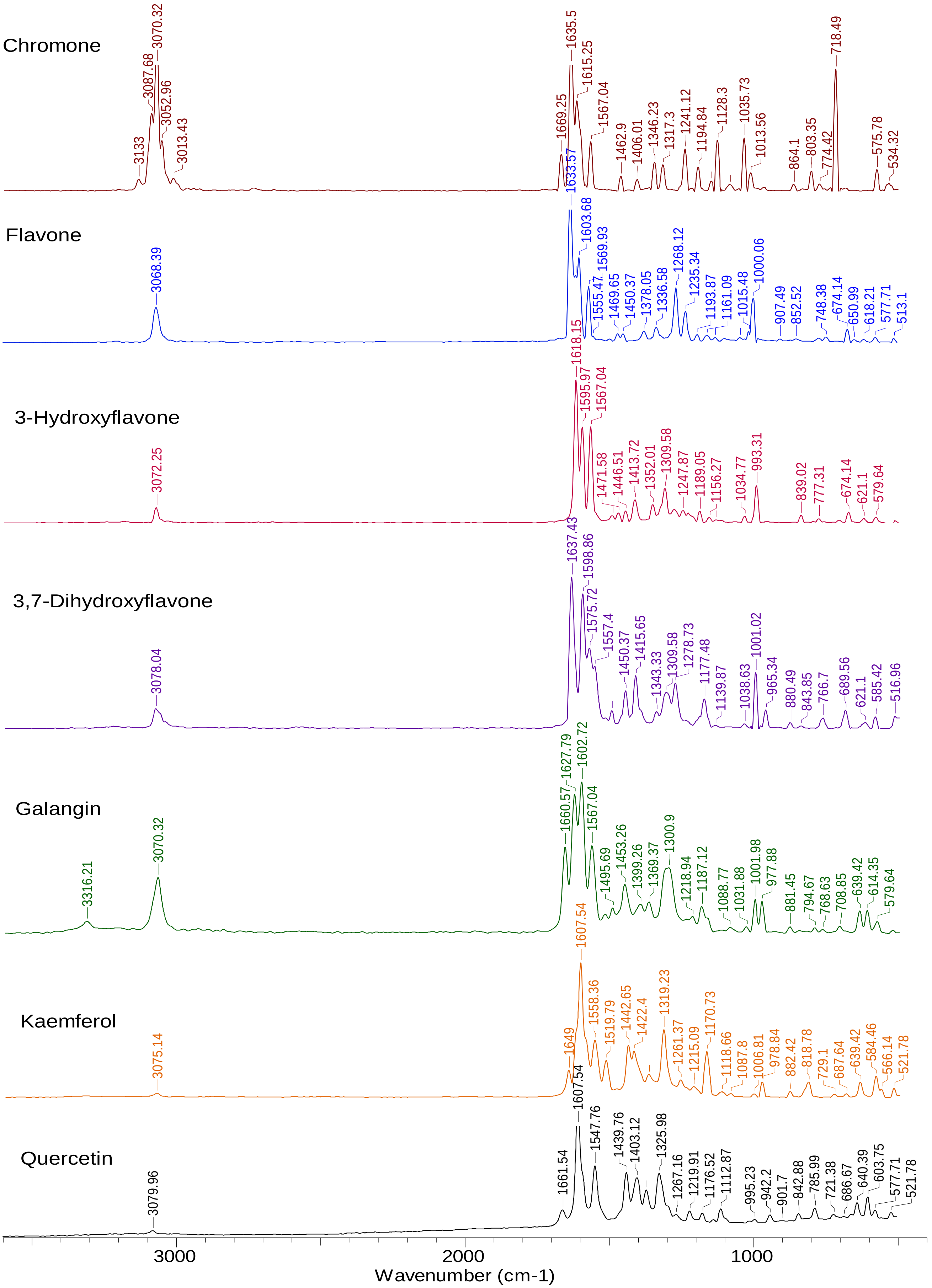

Fig S5. The Raman spectra of chromone derivatives registered in the range of 400-4000 cm^{-1}.

Table S1. Selected bond lengths [Å] and angles [°] of 3-dihydroxyflavone conformers (atom numbering in Fig. 4)

	**Conformers of 3-dihydroxyflavone**	
**Bond lenghts [Å]**	**1**	**2**
C2-C1’	1,476	1,472
C2-C3	1,363	1,363
C3-O3	1,360	1,355
O3-H3	0,965	0,975
C3-C4	1,472	1,458
C4=O4	1,222	1,232
C4-C10	1,475	1,463
C9-C10	1,398	1,400
C8-C9	1,399	1,399
C7-C8	1,384	1,384
C6-C7	1,405	1,406
C5-C6	1,382	1,382
C1’-C2’	1,404	1,403
C2’-C3’	1,490	1,391
C3’-C4’	1,395	1,394
C4’-C5’	1,393	1,394
C5’-C6’	1,404	1,391
C6’-C1’	1,404	1,402
C6-O1	1,363	1,393
O1-C5	1,370	1,369
**Hydrogen bonds [Å]**		
O1…H2’	2,591	2,469
O1…H8	2,557	2,556
O4…H5	2,565	2,633
O3…H3	-	2,057
O2-H6’	2,691	2,397

Table S2. The NBO atomic charges [e] calculated for 3-dihydroxyflavone conformers (atom numbering in Fig. 4)

		**Conformers of 3-dihydroxyflavone**	
**Ring**	**Atom**	**1**	**2**
**A**	C8	-0,235	-0,233
	C9	0,341	0,346
	C10	-0,182	-0,183
	C5	-0,144	-0,144
	C6	-0,214	-0,213
	C7	-0,171	-0,169
**Total charge**		-0,605	-0,596
**C**	O1	-0,489	-0,481
	C2	0,270	0,303
	C3	0,203	0,200
	C4	0,466	0,455
	C10	-0,182	-0,183
	C9	0,341	0,346
**Total charge**		0,609	0,640
**B**	3’C	-0,199	-0,209
	4’C	-0,189	-0,188
	5’C	-0,201	-0,207
	6’C	-0,198	-0,151
	C1’	-0,099	-0,097
	C2’	-0,157	-0,159
**Total charge**		-1,043	-1,011
	O1	-0,489	-0,481
	O3	-0,652	-0,674
	O4	-0,564	-0,619

e = $1{,}602 \cdot 10^{-19}$ C

Table S3. Selected bond lengths [Å] and angles [°] of 3,7-dihydroxyflavone conformers (atom numbering in Fig. 4)

	**Conformers of 3,7-dihydroxyflavone**			
**Bond lenghts [Å]**	**1**	**2**	**3**	**4**
C1'-C7	1,475	1,468	1,468	1,475
C2-C3	1,361	1,366	1,365	1,361
C3-O3	1,360	1,353	1,352	1,359
O3-H3	0,965	0,979	0,978	0,965
C2-C4	1,474	1,462	1,463	1,475
C4=O4	1,223	1,238	1,238	1,223
C4-C10	1,470	1,451	1,450	1,470
C9-C10	1,401	1,403	1,400	1,397
C8-C9	1,395	1,395	1,398	1,398
C7-C8	1,387	1,387	1,388	1,388
C7-O7	1,363	1,362	1,361	1,363
O7-H7	0,963	0,963	0,963	0,963
C6-C7	1,409	1,410	1,410	1,408
C5-C6	1,380	1,378	1,376	1,377
C5-C10	1,405	1,406	1,410	1,408
C1'-C2'	1,404	1,408	1,408	1,404
C2'-C3'	1,390	1,389	1,390	1,390
C3'-C4'	1,395	1,394	1,394	1,396
C4'-C5'	1,393	1,393	1,393	1,393
C5'-C6'	1,393	1,391	1,391	1,393
C6'-C1'	1,404	1,408	1,408	1,404
C9-O1	1,361	1,356	1,356	1,361
O1-C2	1,373	1,376	1,377	1,374
**Hydrogen bonds [Å]**				
O4…H3	-	1,978	1,974	-
O3…H6'	2,678	2,157	2,157	2,688
O1…H2'	2,578	2,309	2,313	2,590
H8…O7	2,581	2,576	2,702	2,704
O1…H8	2,569	2,571	2,551	2,550
O4…H5	2,572	2,653	2,658	2,577

Table S4. The NBO atomic charges [e] calculated for 3,7-dihydroxyflavone conformers (atom numbering in Fig. 4)

		**Conformers of 3,7-dihydroxyflavone**			
**Ring**	**Atom**	**1**	**2**	**3**	**4**
**A**	C8	-0,291	-0,295	-0,322	-0,324
	C9	0,363	0,376	0,377	0,364
	C10	-0,208	-0,208	-0,222	-0,208
	C5	-0,123	-0,122	-0,110	-0,124
	C6	-0,285	-0,284	-0,261	-0,255
	C7	0,344	0,347	0,350	0,347
**Total charge**		-0,200	-0,186	-0,188	-0,200
**C**	O1	-0,489	-0,485	-0,488	-0,492
	C2	0,266	0,311	0,312	0,268
	C3	0,205	0,210	0,198	0,207
	C4	0,467	0,448	0,461	0,465
	C10	-0,208	-0,208	-0,222	-0,208
	C9	0,363	0,376	0,377	0,364
**Total charge**		0,604	0,652	0,638	0,599
**B**	C3’	-0,199	-0,201	-0,201	-0,199
	C4’	-0,190	-0,189	-0,190	-0,190
	C5’	-0,201	-0,206	-0,205	-0,201
	C6’	-0,199	-0,159	-0,159	-0,199
	C1’	-0,093	-0,115	-0,114	-0,098
	C2’	-0,157	-0,174	-0,176	-0,158
**Total charge**		-1,039	-1,044	-1,045	-1,045
	O3	-0,652	-0,679	-0,679	-0,652
	O4	-0,570	-0,634	-0,631	-0,569
	7O	-0,663	-0,660	-0,659	-0,662

e = $1,602*10^{-19}$ C

Table S5. Selected bond lengths [Å] and angles [°] of galangin conformers (atom numbering in Fig. 4)

	Conformers of galangin							
**Bond lenghts [Å]**	**1**	**2**	**3**	**4**	**5**	**6**	**7**	**8**
C3-OH	1,359	1,360	1,360	1,364	1,359	1,350	1,354	1,354
H3-O3	0,965	0,965	0,965	0,966	0,965	0,982	0,977	0,977
C4=O4	1,242	1,242	1,241	1,233	1,219	1,235	1,255	1,256
C5-OH	1,338	1,338	1,338	1,356	1,352	1,351	1,340	1,341
H5-O5	0,993	0,993	0,991	0,966	0,964	0,964	0,985	0,986
C7-OH	1,361	1,361	1,361	1,358	1,362	1,361	1,360	1,360
C1’-C2	1,475	1,475	1,475	1,475	1,475	1,467	1,467	1,467
C2-C3	1,362	1,362	1,362	1,358	1,356	1,361	1,367	1,367
C3-C4	1,465	1,465	1,466	1,472	1,482	1,471	1,453	1,452
C4-C10	1,448	1,448	1,449	1,465	1,476	1,454	1,432	1,433
C10-C5	1,424	1,424	1,427	1,426	1,420	1,423	1,425	1,422
**Hydrogen bonds [Å]**								
C4=O…HO-C5	1,702	1,702	1,710	-	-	-	1,778	1,771
C3-OH…O=C4	-	-	-	-	-	1,920	2,006	2,011

Table S6. The NBO atomic charges [e] calculated for galangin conformers (atom numbering in Fig. 4)

		**Conformers of galangin**							
**Ring**	**Atom**	**1**	**2**	**3**	**4**	**5**	**6**	**7**	**8**
	C8	-0,332	-0,332	-0,363	-0,347	-0,323	-0,352	-0,360	-0,329
	C9	0,380	0,380	0,381	0,383	0,381	0,394	0,393	0,391
	C10	-0,265	-0,265	-0,264	-0,257	-0,255	-0,254	-0,269	-0,270
**A**	C5	0,404	0,404	0,403	0,401	0,408	0,389	0,402	0,398
	C6	-0,352	-0,352	-0,352	-0,352	-0,384	-0,334	-0,321	-0,337
	C7	0,369	0,369	0,369	0,368	0,360	0,360	0,372	0,366
**Total charge**		-0,265	-0,265	-0,264	-0,257	-0,255	-0,254	-0,269	-0,270
**C**	O1	-0,483	-0,483	-0,485	-0,483	-0,489	-0,491	-0,486	-0,481
	C2	0,280	0,280	0,277	0,289	0,261	0,290	0,313	0,327
	C3	0,207	0,206	0,208	0,197	0,210	0,206	0,202	0,198
	C4	0,463	0,463	0,463	0,465	0,463	0,462	0,456	0,455
	C10	-0,265	-0,265	-0,264	-0,257	-0,255	-0,254	-0,269	-0,270
	C9	0,380	0,380	0,381	0,383	0,381	0,394	0,393	0,391
**Total charge**		0,582	0,581	0,580	0,594	0,571	0,607	0,609	0,620
**B**	C3’	-0,198	-0,198	-0,199	-0,204	-0,199	-0,200	-0,208	-0,203
	C4’	-0,188	-0,188	-0,188	-0,190	-0,192	-0,192	-0,186	-0,186
	C5’	-0,201	-0,201	-0,201	-0,204	-0,201	-0,206	-0,206	-0,206
	C6’	-0,198	-0,198	-0,197	0,194	-0,196	-0,163	-0,160	-0,157
	C1’	-0,101	-0,101	-0,101	-0,109	-0,112	-0,099	-0,104	-0,118
	C2’	-0,156	-0,156	-0,156	-0,161	-0,156	-0,167	-0,162	-0,172
**Total charge**		-1,042	-1,042	-1,042	-1,062	-1,056	-1,027	-1,026	-1,042
	O1	-0,483	-0,483	-0,485	-0,483	-0,489	-0,491	-0,486	-0,481
	O3	-0,652	-0,652	-0,651	-0,681	-0,652	-0,680	-0,676	-0,676
	O4	-0,621	-0,621	-0,619	-0,635	-0,541	-0,607	-0,685	-0,687
	O5	-0,670	-0,670	-0,667	-0,657	-0,625	-0,628	-0,665	-0,668
	O7	-0,660	-0,660	-0,658	-0,674	-0,660	-0,660	-0,655	-0,657

e = $1{,}602 \cdot 10^{-19}$ C

Table S7. Selected bond lengths [Å] and angles [°] of kaempherol conformers (atom numbering in Fig. 4)

	**Conformers of kaempherol**				
**Bond lenghts [Å]**	**1**	**2**	**3**	**4**	**5**
C4'-OH	1,363	1,364	1,364	1,364	1,364
C3-OH	1,361	1,357	1,356	1,356	1,356
C4=O	1,243	1,257	1,256	1,256	1,256
C5-OH	1,338	1,341	1,341	1,340	1,340
C7-OH	1,361	1,360	1,360	1,361	1,361
C1'-C2	1,472	1,463	1,463	1,463	1,463
**Hydrogen bonds [Å]**					
C4=O…HO-C5	1,700	1,768	1,771	1,767	1,774
C3-OH…O=C4	-	2,017	2,012	2,018	2,014

Table S8. The NBO atomic charges [e] calculated for kaempherol conformers (atom numbering in Fig. 4)

		**Conformers of kaempherol**				
**Ring**	**Atom**	**1**	**2**	**3**	**4**	**5**
**A**	C8	-0.223	-0.334	-0.349	-0.330	-0.361
	C9	0.380	0.390	0.386	0.390	0.392
	C10	-0.265	-0.262	-0.261	-0.269	-0.268
	C5	0.404	0.396	0.401	0.403	0.402
	C6	-0.353	-0.337	-0.321	-0.351	-0.321
	C7	0.368	0.365	0.367	0.370	0.371
**Total charge**		0.311	0.218	0.223	0.213	0.215
**C**	O1	-0.484	-0.484	-0.488	-0.484	-0.489
	C2	0.286	0.330	0.326	0.333	0.318
	C3	0.200	0.204	0.205	0.190	0.194
	C4	0.464	0.438	0.439	0.453	0.454
	C9	0.380	0.390	0.386	0.390	0.392
	C10	-0.265	-0.262	-0.261	-0.269	-0.268
**Total charge**		0.581	0.616	0.607	0.613	0.601
**B**	C1’	-0.133	-0.147	-0.147	-0.146	-0.133
	C2’	-0.133	-0.149	-0.150	-0.159	-0.138
	C3’	-0.246	-0.247	-0.247	-0.262	-0.287
	C4’	0.329	0.332	0.332	0.327	0.331
	C5’	-0.283	-0.285	-0.285	-0.253	-0.254
	C6’	-0.171	-0.134	-0.134	-0.135	-0.137
**Total charge**		-0.637	-0.630	-0.631	-0.628	-0.618
	O1	-0.484	-0.484	-0.488	-0.484	-0.489
	O3	-0.656	-0.682	-0.680	-0.680	-0.679
	O4	-0.626	-0.692	-0.690	-0.690	-0.688
	O5	-0.670	-0.669	-0.665	-0.668	-0.666
	O7	-0.660	-0.658	-0.657	-0.658	-0.656
	O4’	-0.663	-0.664	-0.664	-0.664	-0.664

e = $1{,}602 \times 10^{-19}$ C

Table S9. Selected bond lengths [Å] and angles [°] of quercetin conformers (atom numbering Fig. 4)

**Bond lenghts [Å]**	**Conformers of quercetin**					
	**1**	**2**	**3**	**4**	**5**	**6**
O3-H3	0,977	0,977	0,977	0,966	0,982	0,965
03-C3	1,355	1,356	1,355	1,360	1,352	1,359
C3-C4	1,449	1,450	1,450	1,463	1,467	1,481
C4=O4	1,256	1,257	1,256	1,246	1,236	1,220
C4-C10	1,434	1,433	1,434	1,449	1,456	1,476
C10-C5	1,422	1,422	1,425	1,424	1,420	1,420
C5-O5	1,340	1,341	1,340	1,338	1,352	1,352
O5-H5	0,987	0,987	0,986	0,993	0,964	0,964
**Hydrogen bonds [Å]**						
H3…O4	2,019	2,012	2,014	-	1,929	-
O4…H5	1,766	1,769	1,772	1,699	-	-
H6…O5	2,589	2,590	2,608	2,582	2,641	2,629
H6…O7	2,684	2,682	2,560	2,685	2,673	2,673

Table S10. The NBO atomic charges [e] calculated for quercetin conformers (atom numbering Fig. 4)

		Conformers of quercetin					
**Ring**		**1**	**2**	**3**	**4**	**5**	**6**
**A**	C8	-0,318	-0,317	-0,350	-0,334	-0,322	-0,324
	C9	0,385	0,387	0,385	0,380	0,393	0,379
	C10	-0,262	-0,270	-0,261	-0,265	-0,254	-0,247
	C5	0,403	0,403	0,402	0,404	0,390	0,388
	C6	-0,351	-0,351	-0,321	-0,350	-0,362	-0,362
	C7	0,366	0,366	0,366	0,368	0,357	0,354
**Total charge**		0,223	0,218	0,221	0,203	0,202	0,188
**C**	O1	-0,491	-0,487	-0,494	-0,490	-0,497	-0,497
	C2	0,314	0,320	0,311	0,283	0,297	0,254
	C3	0,208	0,195	0,210	0,204	0,198	0,214
	C4	0,437	0,453	0,438	0,463	0,460	0,462
	C9	0,385	0,387	0,385	0,380	0,393	0,379
	C10	-0,262	-0,270	-0,261	-0,265	-0,254	-0,247
**Total charge**		0,853	0,868	0,850	0,840	0,851	0,812
**B**	C1’	-0,114	-0,109	-0,113	-0,111	-0,109	-0,107
	C2’	-0,229	-0,201	-0,231	-0,225	-0,234	-0,227
	C3’	0,248	0,274	0,248	0,256	0,248	0,255
	C4’	0,295	0,265	0,294	0,291	0,289	0,287
	C5’	-0,243	-0,271	-0,242	-0,238	-0,242	-0,238
	C6’	-0,160	-0,167	-0,161	-0,202	-0,164	-0,203
**Total charge**		-0,203	-0,209	-0,205	-0,229	-0,212	-0,233
	O1	-0,491	-0,487	-0,494	-0,490	-0,497	-0,497
	O3	-0,679	-0,679	-0,679	-0,653	-0,682	-0,652
	O4	-0,688	-0,691	-0,686	-0,622	-0,610	-0,545
	O5	-0,668	-0,669	-0,665	-0,670	-0,627	-0,624
	O7	-0,659	-0,658	-0,657	-0,661	-0,659	-0,661
	O3’	-0,706	-0,671	-0,706	-0,701	-0,707	-0,703
	O4’	-0,663	-0,699	-0,663	-0,662	-0,666	-0,665

e = 1,602*10^{-19} C

Table S11. The cytotoxic activity of studied compounds in relation to different cancer cell lines

Compound	Parameters	Cell lines	Ref.
Chromone	-	-	-
Flavone	IC50 > 50.000 nM	A549	[1]
3-Hydroxyflavone	LC50=40.4 µM	TIG-1	[2]
	LC50= 64.0 µM	HUVEC	
	IC50= 20 ± 2 µM	B16 melanoma cells/ Lewis lung cells	[3]
3,7-Dihydroxyflavone	GI50=17.4 ± 2.0µM	MCF-7	[4]
	GI50=15.4 ± 1.2 µM	NCI-H460	
	GI50=23.0 ± 1.0 µM	A375-C5	
Galangin	IC50= 48 ± 4 µM	B16 melanoma cells/ Lewis lung cells	[3]
	IC50=275.48 µM	V79	[5]
	IC50=145.0 µmol/L[a]	B16F10	[6]
Kaempferol	LC50=221 µM	TIG-1	[2]
	LC50=167 µM	HUVEC	
	IC50= 51 ± 5 µM	B16 melanoma cells/ Lewis lung cells	[3]
	IC50= 31 ± 8 µM	EAhy 926 endothelial cells	
	IC50=30.92 ±0.05 µM	HepG2	[7]
	IC50=88.02 ±001µM	CT26	
	IC50=70.62 ±0.05 µM	B16F1	
Quercetin	LC50=4.45mM	RAW264.7	[8]
	IC50=118.1±5.55 µM[a]	CT-26	[9]
	IC50=110.7±4.30 µM[a]	LNCaP	
	IC50>120 µM µM[a]	PC3	
	IC50=99.3±6.11 µM[a]	PC12	
	IC50=105.4±5.2 µM[a]	MCF-7	
	IC50=64.9±3.5 µM[a]	MOLT-4	
	IC50=54.3±4.5 µM[a]	U266B1	
	IC50=66.5±3.57 µM[a]	Raji	
	IC50=>120 µM[a]	CHO	
	IC50=50 µM	PC3	[10]
	IC50=50 µM	LNCaP	
	IC50= 35.5±1.1 µM	HeLa	[11]
	IC50=20.9±0.9 µM	NIH-3T3	
	LC50= 303 µM	TIG-1	[2]
	LC50= 61.0 µM	HUVE	
	IC50= 26 ± 4 µM	B16 melanoma cells/ Lewis lung cells	[3]
	IC50= 36 ± 2 µM	EAhy 926 endothelial cells	

[a]for 24h

1. Bible KC, Kaufmann SH. Flavopiridol: a cytotoxic flavone that induces cell death in noncycling A549 human lung carcinoma cells. Cancer Res. 1996;56: 4856–61. Available: http://www.ncbi.nlm.nih.gov/pubmed/8895733
2. Matsuo M, Sasaki N, Saga K, Kaneko T. Cytotoxicity of flavonoids toward cultured normal human cells. Biol Pharm Bull. 2005;28: 253–9. Available: http://www.ncbi.nlm.nih.gov/pubmed/15684479
3. Touil YS, Fellous A, Scherman D, Chabot GG. Flavonoid-induced morphological modifications of endothelial cells through microtubule stabilization. Nutr Cancer. 2009;61: 310–21. doi:10.1080/01635580802521346
4. Neves MP, Cidade H, Pinto M, Silva AMS, Gales L, Damas AM, et al. Prenylated derivatives of baicalein and 3,7-dihydroxyflavone: Synthesis and study of their effects on tumor cell lines growth, cell cycle and apoptosis. Eur J Med Chem. 2011;46: 2562–2574. doi:10.1016/J.EJMECH.2011.03.047

5. Bacanlı M, Başaran AA, Başaran N. The antioxidant, cytotoxic, and antigenotoxic effects of galangin, puerarin, and ursolic acid in mammalian cells. Drug Chem Toxicol. 2017;40: 256–262. doi:10.1080/01480545.2016.1209680
6. Zhang W, Lan Y, Huang Q, Hua Z. Galangin induces B16F10 melanoma cell apoptosis via mitochondrial pathway and sustained activation of p38 MAPK. Cytotechnology. 2013;65: 447–55. doi:10.1007/s10616-012-9499-1
7. Wang J, Fang X, Ge L, Cao F, Zhao L, Wang Z, et al. Antitumor, antioxidant and anti-inflammatory activities of kaempferol and its corresponding glycosides and the enzymatic preparation of kaempferol. Pizzo S V, editor. PLoS One. 2018;13: e0197563. doi:10.1371/journal.pone.0197563
8. Murakami Y, Kawata A, Ito S, Katayama T, Fujisawa S. Radical-scavenging and Anti-inflammatory Activity of Quercetin and Related Compounds and Their Combinations Against RAW264.7 Cells Stimulated with Porphyromonas gingivalis Fimbriae. Relationships between Anti-inflammatory Activity and Quantum Chemical Par. In Vivo. 2015;29: 701–10. Available: http://www.ncbi.nlm.nih.gov/pubmed/26546527
9. Hashemzaei M, Delarami Far A, Yari A, Heravi RE, Tabrizian K, Taghdisi SM, et al. Anticancer and apoptosis-inducing effects of quercetin in vitro and in vivo. Oncol Rep. 2017;38: 819–828. doi:10.3892/or.2017.5766
10. Shenouda NS, Zhou C, Browning JD, Ansell PJ, Sakla MS, Lubahn DB, et al. Phytoestrogens in Common Herbs Regulate Prostate Cancer Cell Growth in Vitro. Nutr Cancer. 2004;49: 200–208. doi:10.1207/s15327914nc4902_12
11. Danihelová M, Veverka M, Sturdík E, Jantová S. Antioxidant action and cytotoxicity on HeLa and NIH-3T3 cells
