# Supplementary material for: Correlations between molecular structure and biological activity in "logical series" of dietary chromone derivatives
Source: PLoS One. 2020 Aug 21;15(8):e0229477. doi: 10.1371/journal.pone.0229477 (PMC7444502; doi:10.1371/journal.pone.0229477)
Supplement: S2 File — (DOCX) [file pone.0229477.s002.docx]

**Table S12. The conformers of 3-hydroxyflavone, 3,7-dihydroxyflavone, galangin, kaempferol and quercetin calculated at B3LYP/6-11++G** level, their energy, dipole moments and depicted intramolecular hydrogen bonding**

| **3-Hydroxyflavone** | |
| --- | --- |
| **(1)** 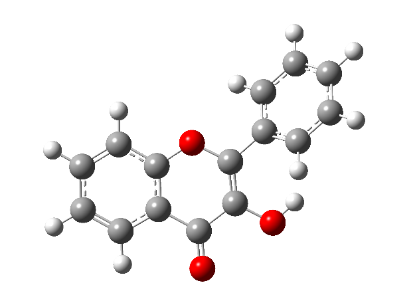 C4  O4 | **(2)** 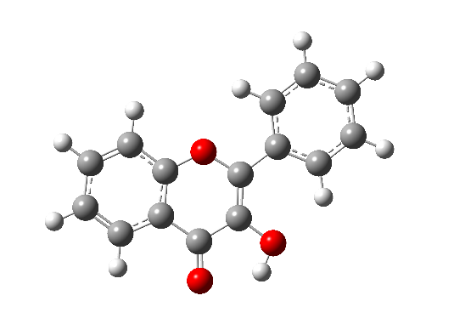 |
| E = -803,494 a.u.= -21 864,193 eV  Dipole moment = 5,5252 D | E = -803,507 a.u.= -21 864,547 eV  Dipole moment = 3,2714 D |
| **3,7-Dihydroxyflavone** | |
| **(1)** 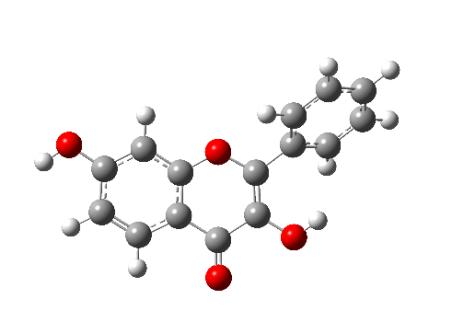 C4  O4  C7  O7  H7  H8  O3  H3  C3 | **(2)** 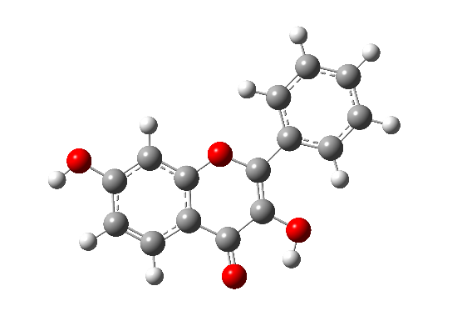 |
| E = -878,743 a.u.= -23 911,824 eV  Dipole moment = 4,3046 D | E = -878,759 a.u. = -23 912,259 eV  Dipole moment = 3,1387 D |
| **(3)** | **(4)** |
| 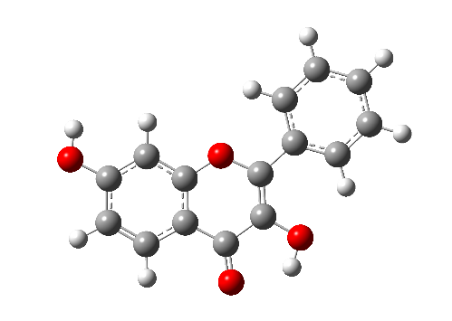  E = -878,758 a.u.= -23 912,232 eV  Dipole moment = 4,2912 D | 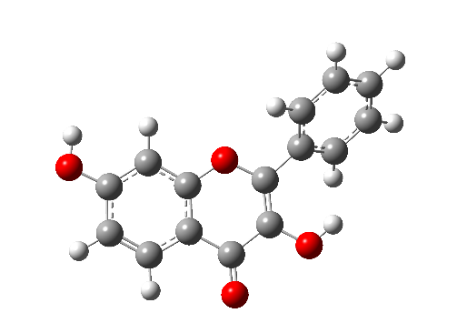 E = -878,743 a.u. = - 23 911,824 eV  Dipole moment = 6,661 D |
| **Galangin** | |
| **(1)** 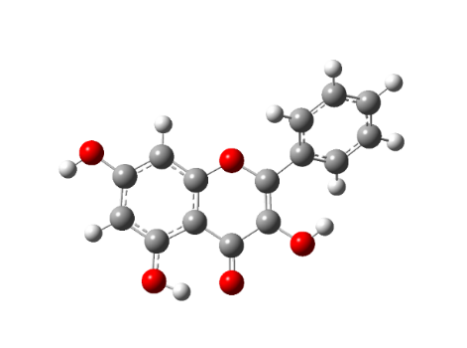 C4  C3  O3  H3  O4  C5  O5 | **(2)** |
| E = -954,00 a.u.= -25 959,67 eV  Dipole moment = 4,9333 D | 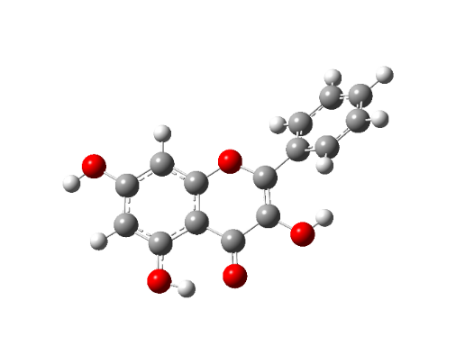 E = -954,00 a.u.= -25 959,67 eV  Dipole moment = 4,9333 D |
| **(3)** 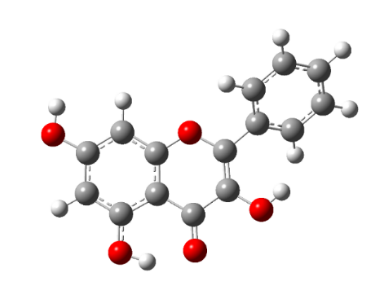 | **(4)** 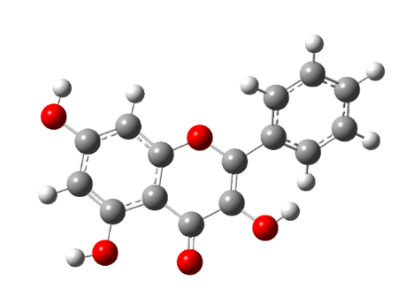 |
| E = -954,00 a.u.= -25 959,67 eV  Dipole moment = 7,5662 D | E = -954,01 a.u.= -25 959,94 eV  Dipole moment = 9,2607 D |
| **(5)** 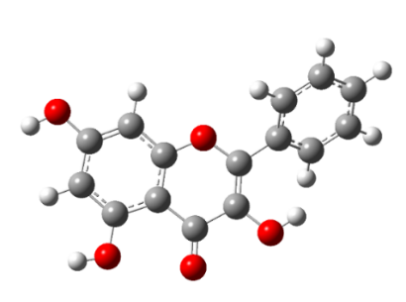 | **(6)** 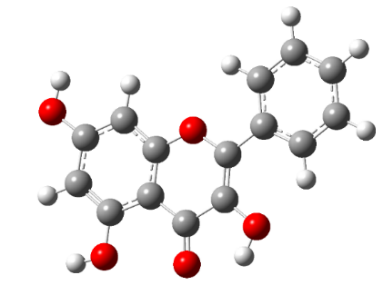 |
| E = -953,98 a.u.= -25 959,13 eV  Dipole moment = 4,3659 D | E = -954,00 a.u.= -25 959,61 eV  Dipole moment = 4,5387 D |
| **(7)** 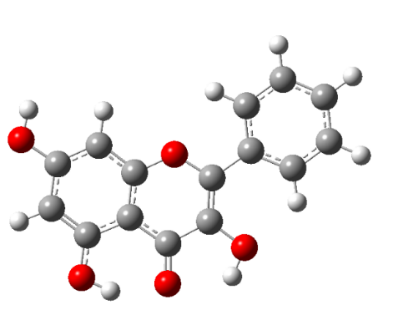 E = -954,017 a.u.= -25 960,13 eV  Dipole moment = 4,6068 D | **(8)** 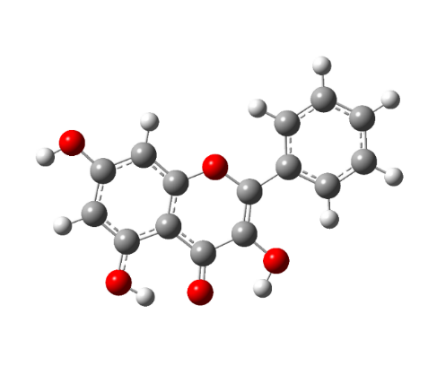 E = -954,018 a.u.= -25 960,16 eV  Dipole moment = 2,3693 D |
| **Kaempferol** | |
| **(1)** 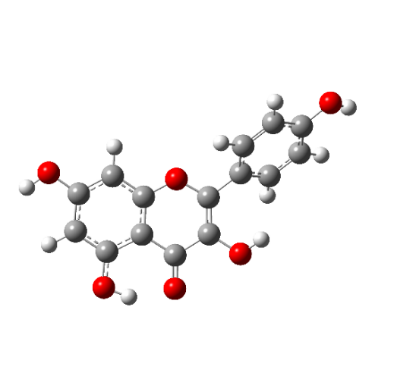 C4  O4  C5  O5  H5  C3  O3  H3  E = -1 029,25 a.u.= - 28 007,32 eV  Dipole moment = 4,8448 D | **(2)** 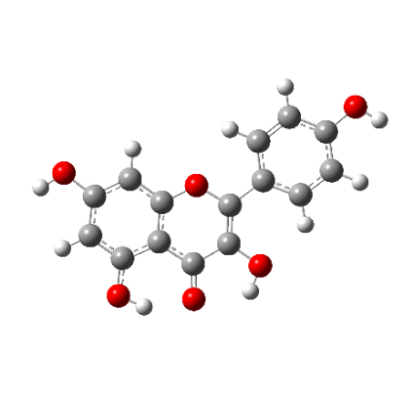 E = -1 029,27 a.u. = - 28 007,87 eV  Dipole moment = 1,7803 D |
| **(3)** 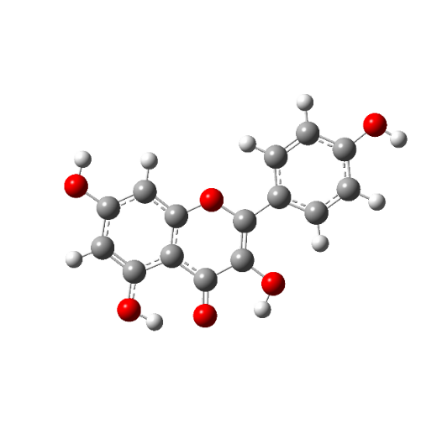 | **(4)** |
| E = -1 029,27 a.u. = - 28 007,87 eV  Dipole moment = 4,4412 D | 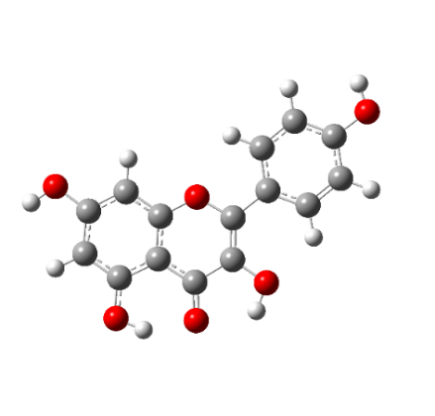 E = -1 029,27 a.u. = - 28 007,87 eV  Dipole moment = 3,8343 D |
| **(5)** |  |
| 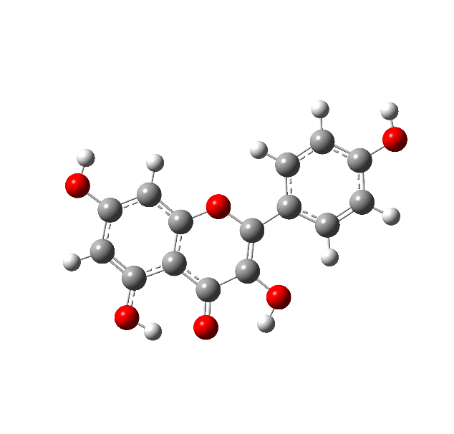 E = -1 029,27 a.u. = - 28 007,87 eV  Dipole moment = 5,9315 D |  |
| **Quercetin** | |
| 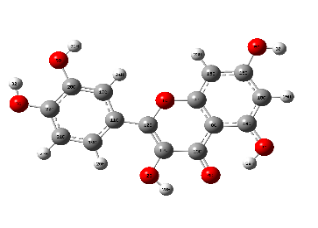 C4  O4  C5  O5  H5  C3  O3  H3  C3’  C4’  C10  **(1)**  E = -1 104.51534 a.u. = -30 055,40 eV  Dipole moment = 4.1316 D | 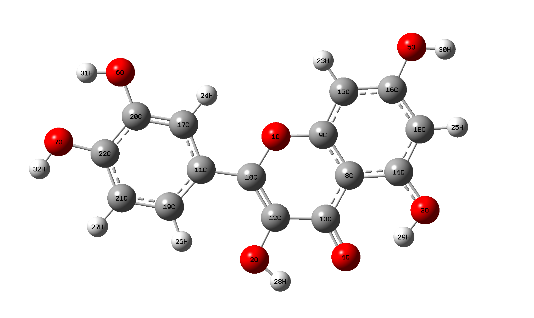 **(2)**  E = -1 104.51478 a.u. = -30 055,39 eV  Dipole moment = 2.7113 D 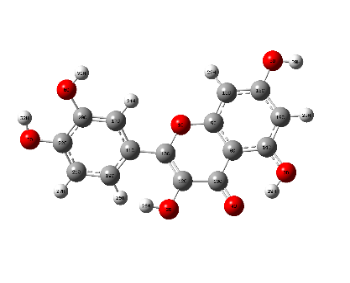 **(4)** |
| 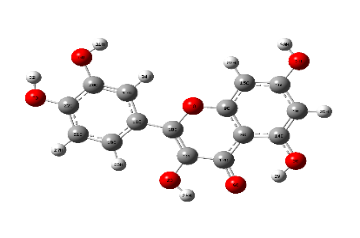 **(3)**  E = -1 104.51430 a.u. = -30 055,38 eV  Dipole moment = 5,6470 D | E = -1 104.50119 a.u. = -30 055,02 eV  Dipole moment = 5,6412 D |
| 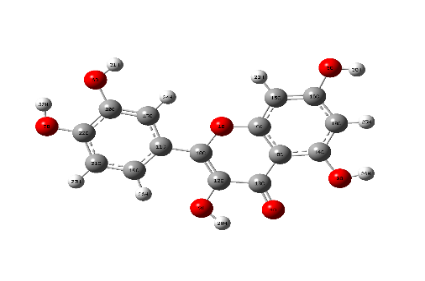 **(5)**  E = -1 104,49471 a.u. = -30 054,84 eV  Dipole moment = 6,5347 D | 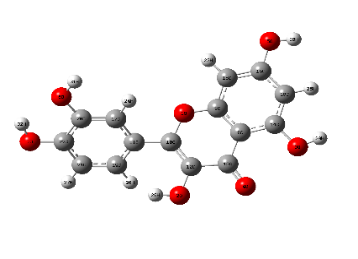 **(6)**  E = -1 104,47753 a.u. = -30 054,38 eV  Dipole moment = 6,0523 D |
